# Using long‐term data from a whole ecosystem warming experiment to identify best spring and autumn phenology models

**DOI:** 10.1002/pei3.10118

**Published:** 2023-06-29

**Authors:** Christina Schädel, Bijan Seyednasrollah, Paul J. Hanson, Koen Hufkens, Kyle J. Pearson, Jeffrey M. Warren, Andrew D. Richardson

**Affiliations:** ^1^ Center for Ecosystem Science and Society Northern Arizona University Flagstaff Arizona USA; ^2^ Woodwell Climate Research Center Falmouth Massachusetts USA; ^3^ School of Informatics, Computing and Cyber Systems Northern Arizona University Flagstaff Arizona USA; ^4^ Environmental Sciences Division and Climate Change Science Institute Oak Ridge National Laboratory Oak Ridge Tennessee USA; ^5^ BlueGreen Labs 9120 Melsele Belgium

**Keywords:** Akaike Information Criterion, climate change, CO_2_, *Larix laricina*, peatland, *Picea mariana*, transition dates

## Abstract

Predicting vegetation phenology in response to changing environmental factors is key in understanding feedbacks between the biosphere and the climate system. Experimental approaches extending the temperature range beyond historic climate variability provide a unique opportunity to identify model structures that are best suited to predicting phenological changes under future climate scenarios. Here, we model spring and autumn phenological transition dates obtained from digital repeat photography in a boreal *Picea*‐*Sphagnum* bog in response to a gradient of whole ecosystem warming manipulations of up to +9°C, using five years of observational data. In spring, seven equally best‐performing models *for Larix* utilized the accumulation of growing degree days as a common driver for temperature forcing. For *Picea*, the best two models were sequential models requiring winter chilling before spring forcing temperature is accumulated. In shrub, parallel models with chilling and forcing requirements occurring simultaneously were identified as the best models. Autumn models were substantially improved when a CO_2_ parameter was included. Overall, the combination of experimental manipulations and multiple years of observations combined with variation in weather provided the framework to rule out a large number of candidate models and to identify best spring and autumn models for each plant functional type.

## Graphical abstract

Here, we leverage multiyear interannual variability in climate with an experimental whole‐ecosystem warming approach to identify model structures that are best suited to predicting phenological changes and future climate scenarios. In spring, seven equally best‐performing models for Larix utilized the accumulation of growing degree days as a common driver for temperature forcing. For Picea, the best two models were sequential models requiring winter chilling before spring forcing temperature is accumulated. In shrub, parallel models with chilling and forcing requirements occurring simultaneously were identified as the best models. Autumn models were substantially improved when a CO_2_ parameter was included.

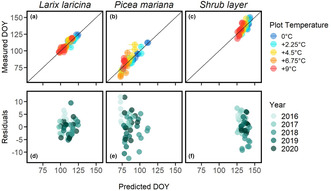

## 1 INTRODUCTION

Rising global temperatures impact ecosystems through a variety of processes, including phenological events such as spring green‐up, flowering, and autumn green‐down (Vitasse et al., 2011). There is substantial evidence that spring green‐up advances with warmer temperatures (Collins et al., 2021; Richardson, Hufkens, et al., 2018) while autumn green‐down (i.e., senescence) is delayed (Richardson, Hufkens, et al., 2018). Together, these changes extend the growing season at both ends. Longer growing seasons impact plant processes such as net annual carbon uptake, growth, and the timing of flowering and are considered a clear indication of a species response to climate change (e.g., Richardson et al., 2013). Further, numerous plant‐related feedbacks to the earth system are driven by phenological events, including seasonal changes in ecosystem photosynthesis, albedo, and partitioning of the surface energy balance (Piao et al., 2019; Richardson et al., 2013; Tang et al., 2016).

Increasing temperature, sufficient winter chilling, and increasing photoperiod are environmental factors that are known to influence bud burst and spring green‐up in many plants. Similarly, leaf senescence and autumn green‐down are triggered by declining temperature, photoperiod, and moisture conditions in the months prior to leaf coloration and/or leaf shedding (Gill et al., 2015; Lang et al., 2019; Liu et al., 2016; Richardson et al., 2013). Which process predominantly influences spring green‐up and autumn green‐down varies among species and there is uncertainty in distinguishing between chilling and photoperiod as cues (Way & Montgomery, 2015). Accurately modeling phenological events for different plant functional types is needed since phenological events directly impact ecosystem functioning and feedbacks to the atmosphere and climate system, and therefore need to be well represented in Earth System Models (Meng et al., 2021).

While numerous process‐based models have been developed to predict spring phenology, many fewer models have been developed for autumn phenology. Most spring models include temperature as the main driver, with parameters often including a starting date and parameters that control the rate of the response to environmental drivers (Basler, 2016). This can be in the form of accumulated growing degree days that need to reach a certain threshold (Wang, 1960; Thermal Time model, e.g., Cannell & Smith, 1983; Hänninen, 1990a) or it could be degree and length of chilling temperature that are required to respond to increasing temperatures in the spring (Parallel Model, Landsberg, 1974; Sequential Model, Hänninen, 1990a; Unified Model, Chuine, 2000).

Autumn green‐down has received less attention than spring green‐up in experimental and modeling studies. However, accurately simulating autumn green‐down is equally important. The main factors involved in driving autumn senescence are decreasing temperature in the form of cold‐degree day (CDD) accumulation (Jeong & Medvigy, 2014) and decreasing daylength (Delpierre et al., 2009). Additionally, dry soils and drought events may advance senescence, however, these factors have not been included in autumn phenology models.

For both spring and autumn, more complex phenology models come at the cost of more parameters, and in general these more complex models have not proven to perform much better than models with fewer parameters (Basler, 2016). Therefore, while more complexity makes the models more flexible, and thus more likely to fit the observational data, it does not mean the models do better at representing the underlying processes or generalizing well in time and space. Careful interpretation of data model fits is required as good data fits are not always related to the underlying biologically relevant responses (Hunter & Lechowicz, 1992). However, Akaike's Information Criterion (AIC) offers an objective path to model selection that balances complexity against goodness of fit (Burnham & Anderson, 2004), and which has been commonly applied to phenological models.

Accurate modeling of phenological events is critical to improve the coupling between the atmosphere and the biosphere in Earth System Models. Outstanding questions remain, however, about how existing phenological models perform with interannual climate variability and with future climate scenarios including extreme climate conditions (Piao et al., 2019). Here, we utilize multiyear interannual variability in weather, and an experimental treatment that far exceeds historical variability to test models under a wide range of environmental conditions. We use the long‐term multifactor global warming experiment called “Spruce and Peatland Responses Under Changing Environments” (SPRUCE, Hanson et al., 2017), over which interannual variation in weather over five years is superimposed, to identify spring and autumn models that best describe phenological events for the three plant functional types (*Larix*, *Picea*, shrubs) that characterize the SPRUCE ecosystems. Phenological data from SPRUCE provide an ideal framework to challenge model capabilities and to isolate drivers influencing spring and autumn plant phenology (Hänninen et al., 2019; Prevéy et al., 2021). Additionally, because the multifactor experiment includes elevated atmospheric CO_2_ as a treatment, this offers the potential to consider how elevated CO_2_ influences phenological transitions, and whether CO_2_ needs to be included in phenological models.

Here, we explore the following questions: (1) How well do existing spring and autumn models predict phenological transition dates at the SPRUCE experiment? (2) Can we identify key mechanisms and drivers for different species that drive spring green‐up and autumn green‐down? (3) Do the “best” models fitted to the experimental data generalize well and make good phenological predictions at other sites; and (4) Is there evidence that elevated CO_2_ plays a role in in either spring or autumn phenology?

## 2 MATERIALS AND METHODS

### 2.1 Experimental site description

For this modeling study, we used vegetation phenology data from the “Spruce and Peatland Responses Under Changing Environments” (SPRUCE) experiment (Hanson et al., 2017). This whole ecosystem warming experiment is a long‐term, multifactor experiment located in Minnesota, USA, at the S1 bog (47° 30.476′ N; 93° 27.162′ W; 418 m above mean sea level [Kolka et al., 2011]). Mean annual temperature is 3.4°C (1961–2009) and mean annual precipitation is 780 mm (Sebestyen et al., 2011). The ombrotrophic peat bog provides hummock and hollow microtopography, with a perched water table of 10–20 cm above the hollows after snowmelt and commonly −20 to −30 cm below hollows in midsummer (Iversen et al., 2018). The dominant woody vegetation includes *Picea mariana*, (Mill.) B.S.P. (black spruce), *Larix laricina* (Du Roi) K. Koch (*Larix*), and a mixture of ericaceous shrubs such as *Rhododendron groenlandicum* (Oeder) Kron & Judd (Labrador tea), *Chamaedaphne calyculata* (L.) Moench. (leatherleaf), and other species. A detailed description of the experimental setup and sustained temperature and elevated CO_2_ treatment can be found in Hanson et al. (2017).

Briefly, the experiment consists of 10 large open‐top enclosures (12.8 m diameter, 7 m tall) of an octagonal shape and transparent greenhouse panels. Five levels of ecosystem warming are applied (nominally 0°, +2.25°, +4.5°, +6.75°, and + 9°C) year‐round and half the enclosures receive elevated CO_2_ during the growing season (~500 pmm above ambient enclosures [Hanson et al., 2017]). Air warming was achieved by heating the air to a height of ~6 m in each enclosure using propane‐fired heat exchangers and a system of blowers and conduits (Hanson et al., 2017). Deep‐soil heating consists of arrays of 3 m deep vertical low‐wattage heating elements that are installed in circles around and within each enclosure. Ecosystem warming occurs year‐round with deep‐soil heating starting in June 2014, air warming in August 2015 (Hanson et al., 2016), and CO_2_ fumigation beginning in June 2016 (Hanson et al., 2021).

### 2.2 PhenoCam imagery

**FIGURE 1:**
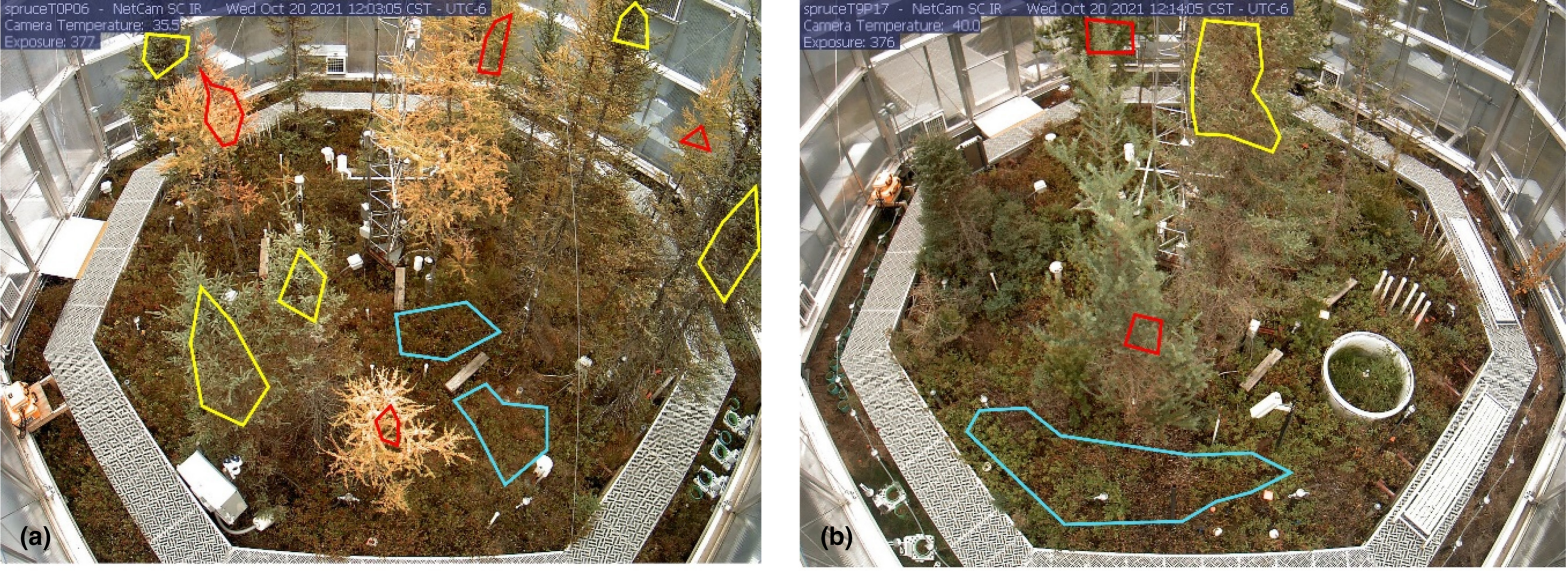
Camera view in a 0°C enclosure (a) and a + 9°C enclosure (b) on October 20, 2021. Polygons show regions of interest for larch (red), spruce (yellow), and shrub (blue). Note the yellow larch needles in (a) while (b) still has green larch needles.

Digital imagery was used to track seasonal vegetation greenness in each enclosure. One digital camera (NetCam model SD130BN, StarDot Technologis; Richardson et al., 2007; Richardson, Hufkens, et al., 2018) was installed in each enclosure at a height of 6 m above the ground on the south facing wall of the enclosure. Images are taken in each plot every 30 min from 4:00 to 22:00 throughout the entire year and are uploaded in near‐real time to the PhenoCam server (https://phenocam.nau.edu/webcam/) for archiving and processing (Figure 1). For each camera field of view, three separate regions of interest (ROI) were defined which correspond to (1) *Larix* (plant functional type DN, deciduous needleleaf), (2) *Picea* (plant functional type EN, evergreen needleleaf), and (3) a mixed shrub layer (plant functional type SH). Transition dates corresponding to the start of the “greenness rising” (spring) and end of the “greenness falling” (autumn) were used for the period of 2016–2020 and were derived from the three‐day smoothed Green Chromatic Coordinate (G_cc_) based on the 25% seasonal amplitude threshold (archived dataset in (Schädel, Richardson, et al., 2021)). The two tree species, *Larix* and *Picea*, can be classified as deciduous broadleaf conifer, and evergreen needleleaf conifer functional types, both common to the circumpolar polar region. The shrub layer consists of evergreen and deciduous shrubs and while the Phenocam approach cannot distinguish the different species within a single mask across the growing season, we have species‐specific ground observations of green‐up and green‐down that align with the Phenocam results (Richardson, Latimer, et al., 2018; Schädel et al., 2019, 2020; Schädel, Pearson, et al., 2021).

We visually identified snow on trees and on the ground from each camera in mid‐day images throughout the entire dataset and excluded these days from analysis (details in (Schädel, Richardson, et al., 2021)). The blue‐white of snow adds noise to the underlying G_cc_ signal which is shifted downward on days with snow (Seyednasrollah et al., 2021). A more detailed description of image analysis and data processing is documented in Richardson, Hufkens, et al. (2018).

For EN vegetation, seasonal changes in G_cc_ are driven more by changes in leaf‐level pigments than they are by the production and senescence of foliage and changes in leaf area, but transition dates derived from the G_cc_ time series have been shown to correlate well (r values >0.9) with the switching “on” and “off” of photosynthetic activity (Seyednasrollah et al., 2021). For DN vegetation, the spring transition dates align with budburst and the production of new foliage, while autumn transition dates are driven by the timing of senescence and leaf shedding. For SH vegetation, while there is some green‐up of residual overwintered foliage, the spring transition dates align well with bud break in the dominant shrub species *Rhododendron groenlandicum* and *Chamaedaphne calyculata*.

### 2.3 Environmental data

On a plot level, environmental monitoring includes half‐hourly air‐ and soil‐temperature measurements, water table depth, relative humidity, photosynthetically active radiation, soil water content, and wind speed. Air temperature used in this modeling analysis was averaged across two measured points in each enclosure at a height of 2 m in 30‐minute intervals (data available at http://sprucedata.ornl.gov). Daily mean values were calculated for each enclosure. Soil temperature from two locations within the main board walk area in each enclosure was measured in 30‐minute intervals at nine depths (0, 5, 10, 20, 30, 40, 50, 100, and 200 cm) and daily averages were calculated for all depths. Water table depth was measured daily within a well in each enclosure. Depth to the water table is referenced to the estimated mean hollow height in each enclosure (Norby et al., 2019).

### 2.4 Modeling framework

We applied 19 spring and 10 autumn models using the R package *phenor*, a modeling framework developed and described by Hufkens et al. (2018). The package was specifically developed to use vegetation phenology data from the PhenoCam network (among other phenology datasets), combined with location‐specific climate data obtained from the Daymet dataset (Thornton et al., 2020) using the R package *daymetr*. Together, these R packages provide functions to compare multiple models and additionally provide the environment to easily add new models (we note that we expanded the range of standard autumn models by four which have been incorporated into the *phenor* codebase). Using predefined parameter ranges with a uniform distribution (i.e., noninformative Bayesian priors), we used simulated annealing for parameter optimization, similar to previous studies (Chuine et al., 1998; Melaas et al., 2013; Richardson & O'Keefe, 2009).

Spring and autumn models are listed in Table 1 along with their drivers and number of parameters. The models represent different assumptions about the underlying drivers and mechanisms controlling phenological transitions. The mathematical representation of these processes provides a wide range of model structures (for details see appendices in Basler, 2016; Hufkens et al., 2018) (see to be tested against the data). As described above, the extreme combination of temperature and CO_2_ treatments at SPRUCE, as well as the underlying interannual variability, provides a unique opportunity to test phenological models.

**TABLE 1:** Details of spring and autumn models adapted from Basler et al. (2016) and Hufkens et al. (2018).

Model name	Abbreviation	Drivers	# parameters	Reference/comments
Spring				
Linear	LIN	F	2	Linear regression and temperature
Thermal Time	TT	F	3	(Cannell & Smith, 1983; Hänninen, 1990b; Hunter & Lechowicz, 1992)
Thermal Time sigmoid	TTs	F	4	
Photo‐thermal time	PTT	PF	3	(Črepinšek et al., 2006; Masle et al., 1989)
Photo‐thermal time sigmoid	PTTs	PF	4	
M1	M1	PF	4	(Blümel & Chmielewski, 2012)
M1 sigmoid	M1s	PF	5	
Alternating	AT	CF	5	(Cannell & Smith, 1983; Murray et al., 1989)
Sequential	SQ	CF	8	(Hänninen, 1990b; Kramer, 1994)
Sequential b	SQb	CF	8	
Sequential M1	SM1	CPF	9	Combination of Sequential and M1 model
Sequential M1b	SM1b	CPF	9	
Parallel	PA	CPF	9	(Hänninen, 1990b; Kramer, 1994; Landsberg, 1974)
Parallel b (bell‐shaped)	PAb	CPF	9	
Parallel M1	PM1	CPF	10	Combination of Parallel and M1 model
Parallel M1b (bell‐shaped)	PM1b	CPF	10	
Unified M1	UM1	CPF	9	(Chuine, 2000)
Growing season index	SGSI	FPV	9	(Xin et al., 2015)
Growing season index	AGSI	FPV	9	(Xin et al., 2015)
Autumn				
Chilling degree day	CDD	C	3	(Jeong & Medvigy, 2014)
Chilling degree day and CO_2_	CDDCO_2_	C	4	(Jeong & Medvigy, 2014)
Chilling degree day sigmoid	CDDs	C	4	(Jeong & Medvigy, 2014)
Chilling degree day sigmoid and CO_2_	CDDsCO_2_	C	5	(Jeong & Medvigy, 2014)
Chilling degree day photoperiod	CDDP	CP	3	(Jeong & Medvigy, 2014)
Chilling degree day photoperiod and CO_2_	CDDPCO_2_	CP	4	(Jeong & Medvigy, 2014)
Chilling degree day water table	CDDM	CM	4	(Jeong & Medvigy, 2014)
Chilling degree day water table and CO_2_	CDDMCO_2_	CM	5	(Jeong & Medvigy, 2014)
Photoperiod water table	PPM	PM	3	(Jeong & Medvigy, 2014)
Photoperiod water table and CO_2_	PPMCO_2_	PM	4	(Jeong & Medvigy, 2014)

*Note*: Models are grouped by drivers: forcing temperature (F), chilling temperature (C), vapor pressure deficit (V), and moisture (M). Shading groups models with the same base structure together.

The spring models range from a simple regression (Linear model) using two parameters to more complex nonlinear models including chilling requirements, forcing temperatures, and photoperiod (Parallel model) with 10 parameters. All spring models include some form of temperature influence upon spring green‐up (e.g., chilling or forcing temperature) and some models include photoperiod (Table 1, Basler et al. 2016).

Few autumn models exist, and we added four autumn models to the *phenor* framework that included processes that might drive senescence. Based on previous studies, we hypothesized that: (1) warmer temperatures delay senescence, (2) photoperiod acts as a trigger or driver, (3) drier soils advance senescence, and (4) higher atmospheric CO_2_ concentrations accelerate senescence. Each of the autumn models contained one or more of these processes. In the original chilling degree day (CDD) model, leaf senescence occurs when the amount of CDD is larger than a certain species‐specific threshold (Jeong & Medvigy, 2014). The CDDP model is adapted from the PTT model (spring thermal time model with photoperiod) and includes a photoperiod variable (daylength in hours per day based on location) in the chilling requirement. We added two autumn models that incorporated water table depth to the fall green‐down criterion (CDDM, PPM). The CDDM model included water table depth in the chilling requirement while the PPM model only used water table depth for the senescence criterion and no temperature forcing at all. A second version of each autumn model included a CO_2_ parameter.

We used the function *pr_fm_phenocam* to format the PhenoCam data into a flattened nested list suitable for model comparison. We extracted the 90th percentile of the G_cc_ over a three‐day window and estimated transition dates at the 25% threshold of the seasonal amplitude of greenness. Traditionally, *phenor* uses climate data from Daymet and we replaced Daymet temperatures with enclosure‐specific air and soil temperature datasets to account for the experimental temperature treatment. Water table depth was added as an additional environmental variable. All models were fitted using the function *pr_fit* and parameter optimization was run using generalized simulated annealing (GenSA). The same upper and lower limits for parameter ranges for all plant functional types (Table S1) were provided by Hufkens et al. (2018). We ran the code in 25 parallel chains for each 40,000 iterations and all model fits were performed on the high‐performance computing cluster “Monsoon” at Northern Arizona University.

Model output provides predicted transition dates, parameter values, root mean square error (RMSE), and AIC. The AIC is an estimator of model prediction error and is used for model selection. AIC is calculated as AIC = 2 k + n(log(sum(measured − predicted)^2)/n), where n is length of measured data, and k represents the number of model parameters. This AIC calculation is a custom function that accepts loess regression. To identify the “best” model in each season, we selected the model with the lowest AIC across 25 parallel model chains and 19 models in spring and 10 models in autumn. We considered models with ∆AIC <2 to be essentially equivalent in terms of performance, whereas models with ∆AIC ≥2 had little support and models with ∆AIC ≥10 no support (Burnham & Anderson, 2004).

### 2.5 Statistical analyses

We performed linear mixed effects models to assess the relationship of temperature and CO_2_ and their interaction with spring and fall transition dates using the R package *nlme* (Pinheiro et al., 2012). Mean annual differential plot air temperature and CO_2_ were fixed effects and year was treated as a random effect. We used the mean annual measured temperature differential between enclosures and the two unheated control enclosures for each plot. Statistical analyses were performed using R Version 4.0.5.

### 2.6 Model validation using other sites

We performed model validation to ensure that models can be used to extrapolate in space and time and are not simply overfit to the data from the SPRUCE experiment. We selected Phenocam sites with similar vegetation to the SPRUCE experiment. For *Larix* and *Picea*, we selected the BOREAS Southern Old Black Spruce study area in Prince Albert National Park, Saskatchewan, Canada (Suppl. Figure S1). For both plant functional types, there were nine years of data available (2012–2020) from the “canadOBS” PhenoCam. For the shrub layer, we selected the Mer Bleue Conservation Area in Ottawa, Canada, which has a similar composition of plant species as the shrub layer in the SPRUCE experiment (Suppl. Figure S1). There were eight years of data available for the shrub layer (2013–2020) from the “merbleue” PhenoCam. We used the optimal parameter values determined for SPRUCE in the validation analysis. For model evaluation, we excluded autumn models for “canadaOBS” or “merbleue” with water table depth due to the lack of available data.

## 3 RESULTS

### 3.1 Changes in transition dates with warming

**FIGURE 2:**
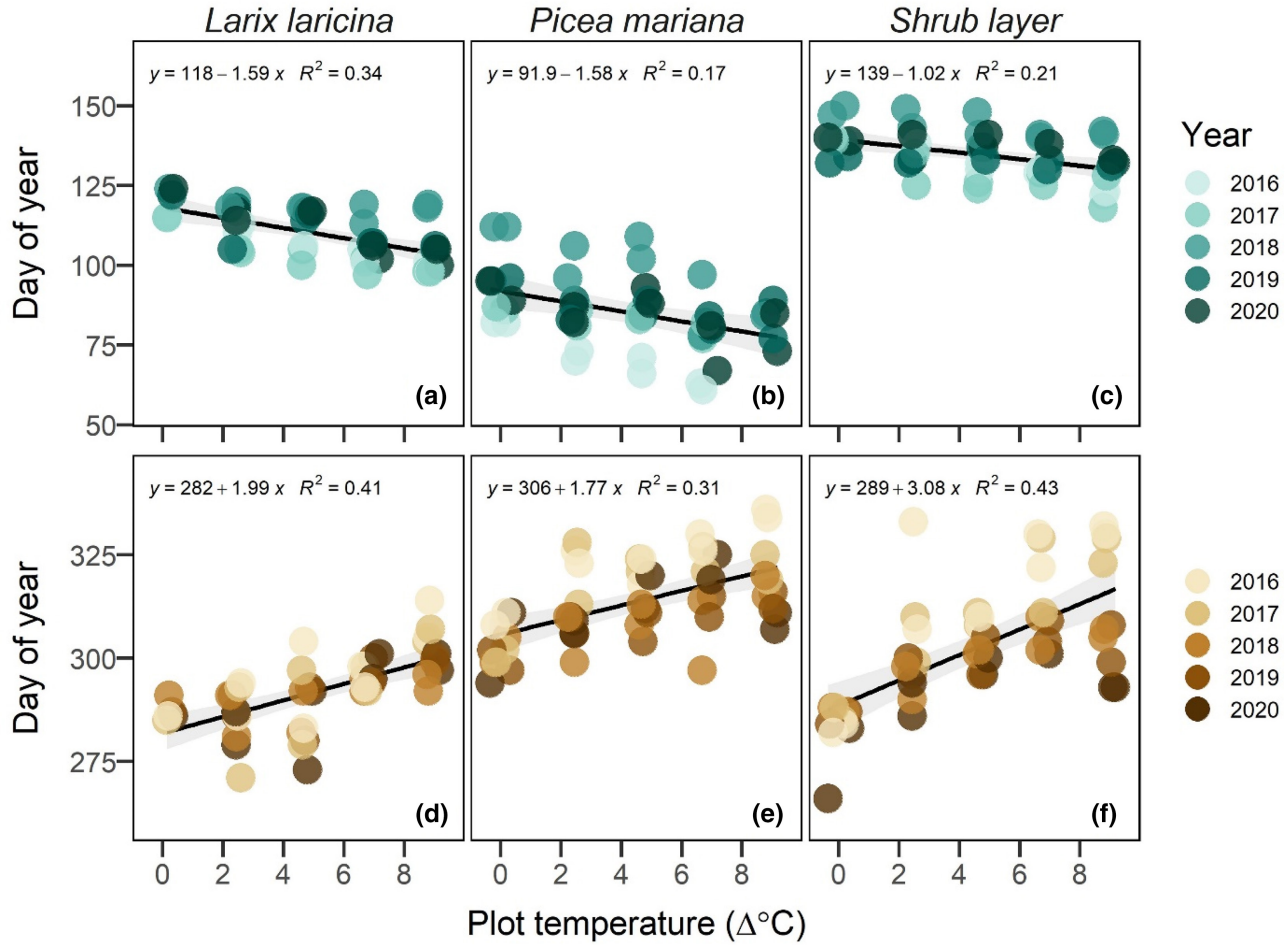
Transition dates obtained from digital camera imagery in response to whole‐ecosystem warming for spring green‐up (a–c) and autumn green‐down (d–f) for five years. Plot temperature is the mean annual measured 2 m air temperature differential between enclosures and the two unheated control enclosures for each plot.

**FIGURE 3:**
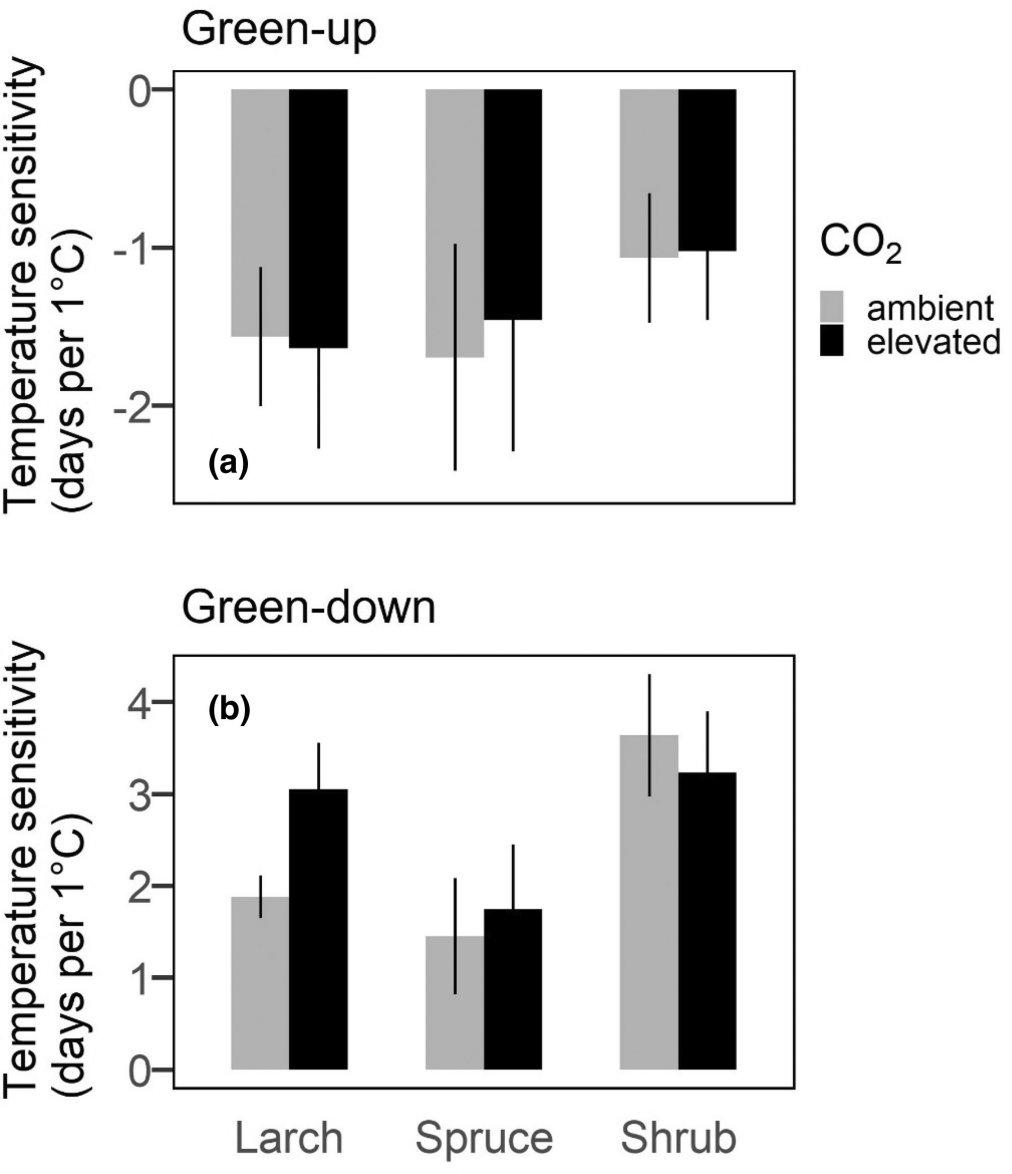
Temperature sensitivity expressed in days per degree Celsius for each functional plant type for green‐up (a) and green‐down (b).

Ecosystem warming significantly advanced spring green‐up. Temperature sensitivity, a metric to quantify the change in phenology per degree change in temperature, averaged (mean ± 1 SD) −1.1 ± 0.6 days per degree Celsius across all plant functional types and years (Figures 2 and 3, Table 2, calculated here as the mean annual 2 m air temperature differential relative to unheated controls). Consequently, spring green‐up occurred about 10 days earlier in the +9°C warming enclosures, relative to the unheated control enclosures. Autumn green‐down was delayed by 2.1 ± 2.4 days per degree warming across plant functional types and years and thus green‐down occurred almost three weeks later in the +9°C warming enclosures, relative to the unheated control enclosures. Together, the effects of advanced spring green‐up and delayed autumn senescence resulted in the overall period of vegetation activity being extended by 3.2 ± 2.4 days per degree, or almost 30 days in the warmest enclosures.

**TABLE 2:** Linear mixed effects model results by plant functional type to explain variation in spring green‐up and autumn green‐down.

Season	Species	Fixed effects	Estimate	SE	Df	*t* value	*p* value
Spring	*Larix laricina*	Intercept	118.09	2.87	39	41.11	<.001
		Temperature	−1.59	0.19	39	−8.30	<.001
	*Picea mariana*	Intercept	92.69	4.83	40	19.2	<.001
		Temperature	−1.94	0.29	40	−6.69	<.001
	Shrub layer	Intercept	139.39	2.77	43	50.26	<.001
		Temperature	−1.0	0.16	43	−6.34	<.001
Autumn	*Larix laricina*	Intercept	286.56	1.82	37	157.76	<.001
		CO_2_	−16.94	3.02	37	−5.61	<.001
		Temperature	1.86	0.29	37	6.46	<.001
		CO_2_ x Temperature	1.38	0.50	37	2.75	.01
	*Picea mariana*	Intercept	307.85	3.37	43	91.44	<.001
		CO_2_ Temperature	−4.22	1.63	43	−2.58	.01
			1.77	0.26	43	6.82	<.001
	Shrub layer	Intercept	288.38	4.47	43	64.5	<.001
		Temperature	3.06	0.38	43	8.12	<.001

*Note*: Only final models are shown after model selection. CO_2_ got dropped in the final spring models and in the shrub autumn model. SE is Standard error and DF degrees of freedom.

The temperature sensitivity of spring green‐up was similar across species. However, in autumn, the response to warming was stronger (3.4 days delay per 1°C warming) for the shrub layer than for *Larix* (two days delay per 1°C warming) or *Picea* (1.6 days delay per 1°C warming).

The effects of elevated CO_2_ on vegetation phenology differed across seasons and to a lesser degree among plant functional types. There was no measurable effect of elevated CO_2_ treatments on spring green‐up of any of the three plant functional types (Table 2). By comparison, elevated CO_2_ significantly advanced autumn senescence compared to ambient conditions by an average (across all temperature treatments) of −16.9 ± 3.0 days in *Larix* and − 4.2 ± 1.6 days in *Picea* (Table 2). Additionally, there was a positive interaction with temperature and CO_2_ in *Larix* in autumn, meaning that temperature sensitivity was stronger with elevated CO_2_ than under ambient CO_2_ conditions (Figure 3b, Table 2). Shrub layer autumn senescence was advanced by −3.0 ± 4.2 days, but this effect was not statistically significant (*p* value = .49).

### 3.2 Spring models

**FIGURE 4:**
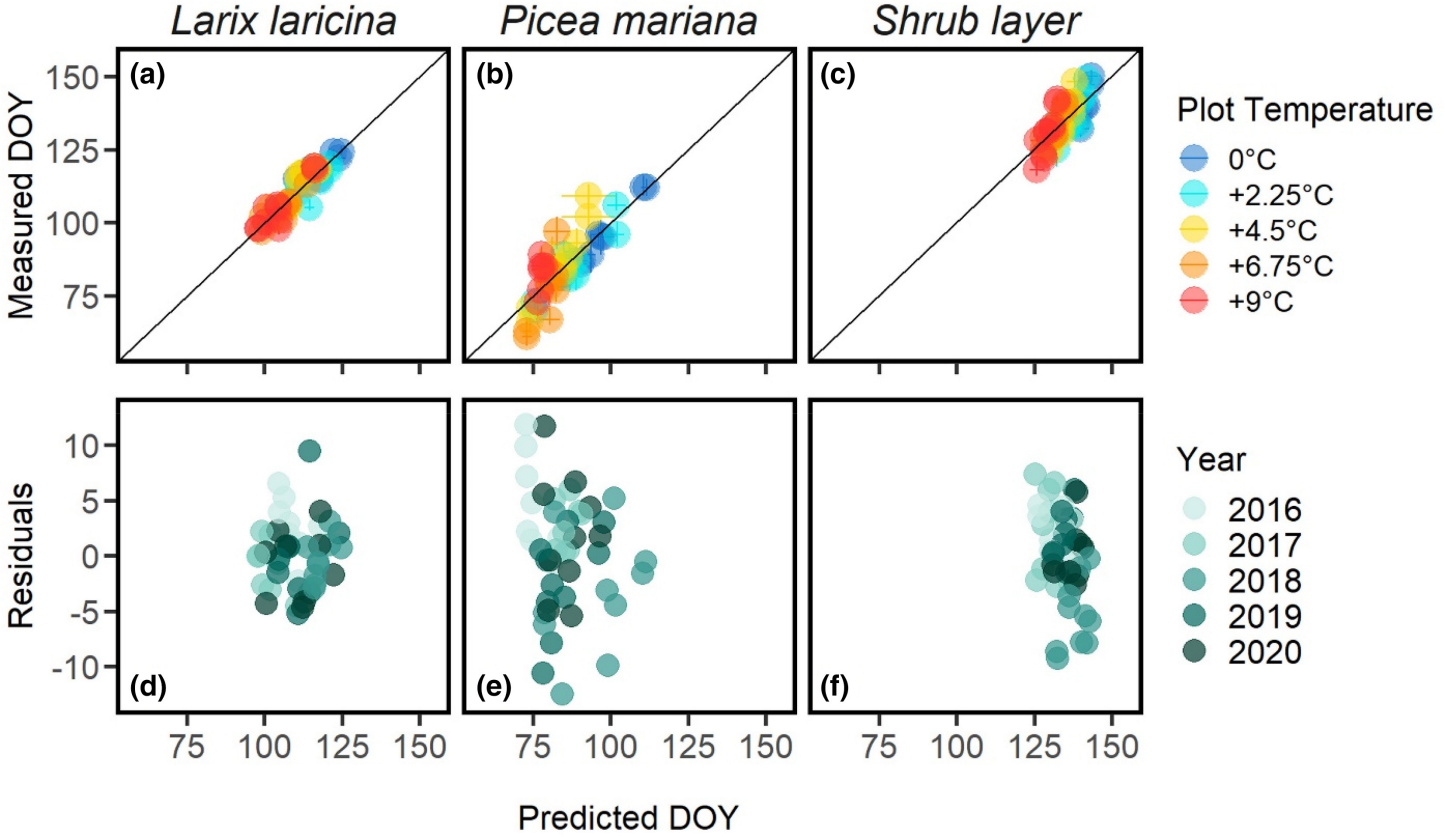
Measured versus predicted day of year (DOY) for spring green‐up showing results from the best spring model of the 25 parallel chains based on lowest Akaike Information Criterion (AIC) for (a) larch—SM1b model, (b) spruce—SM1 model, and (c) shrub—PAb model. Residuals and predicted DOY for larch (d), spruce (e), and the shrub layer (f). In (a)–(c) different colors represent targeted differential plot temperatures and in (d)–(f) different colors represent different calendar years. Air temperature was used for all models. Variation in (a)–(c) shows upper and lower 90% confidence interval on the measured DOY transition dates and standard deviation for predicted DOY transition dates.

Spring green‐up transition dates in all plant functional types and across all levels of warming were very well fitted by at least a couple of the 19 spring models provided in the *phenor* R package (Figure S1, Table S2). Model residuals were symmetrical for the selected models and did not show any clear patterns among years or in relation to temperature treatments (Figure 4d–f). Using AIC for model selection allowed us to account for the complexity of models to identify the best model for each plant functional type. By comparison, RMSE is not ideal for model selection as it does not account for greater flexibility (more degrees of freedom, and hence likely better fit) or more complex or more highly parameterized models.

For *Larix*, we identified seven models (M1, SM1, SM1b, TT, TTs, PTT, PTTs) that fit the data equally well with a ∆AIC <2 from the lowest AIC (Figure 4a, Table S2). These models are all quite similar in structure. The M1, TT, TTs, PTT, and PTTs model all include accumulation of growing degree days for temperature forcing while the PTT and PTTs models additionally include a photoperiod factor (TTs and PTTs use a sigmoidal temperature response rather than linear). The M1 model expands the TT model by adding an exponential constant to account for photoperiod. The SM1 and SM1b (using bell‐shaped chilling function) models are M1 models with the sequential component that chilling requirements must be fulfilled before forcing temperature is accumulated (Table 1). Even though the SM1 and SM1b models are complex models with eight to nine parameters, they perform well and have a low RMSE of 3.3–3.6 days.

For *Picea*, both sequential models with a photoperiod term (SM1 and SM1b) are distinctly better than the other models (Table S2, RMSEs of 7.5) and most other models have a ∆AIC > 10 and therefore no support. Interestingly, SQ and SQb, which are both sequential models but without the photoperiod term, performed poorly. Thus, in contrast to our results for *Larix*, for *Picea* we were able to substantially narrow the pool of candidate models to just two models that are well‐supported by the observational data.

The shrub layer showed both parallel models (PA and PAb, the latter with a bell‐shaped chilling function) to be distinctly better than any other model (Figure 4c, Table S2). In the parallel models, chilling and forcing requirements are occurring simultaneously rather than in sequence. Given that all other models had a ∆AIC > 10, they are either too complex or missing key processes. Thus, similar to *Picea*, we were able to narrow down the set of candidate models to just two models in shrub.

### 3.3 Autumn models

**FIGURE 5:**
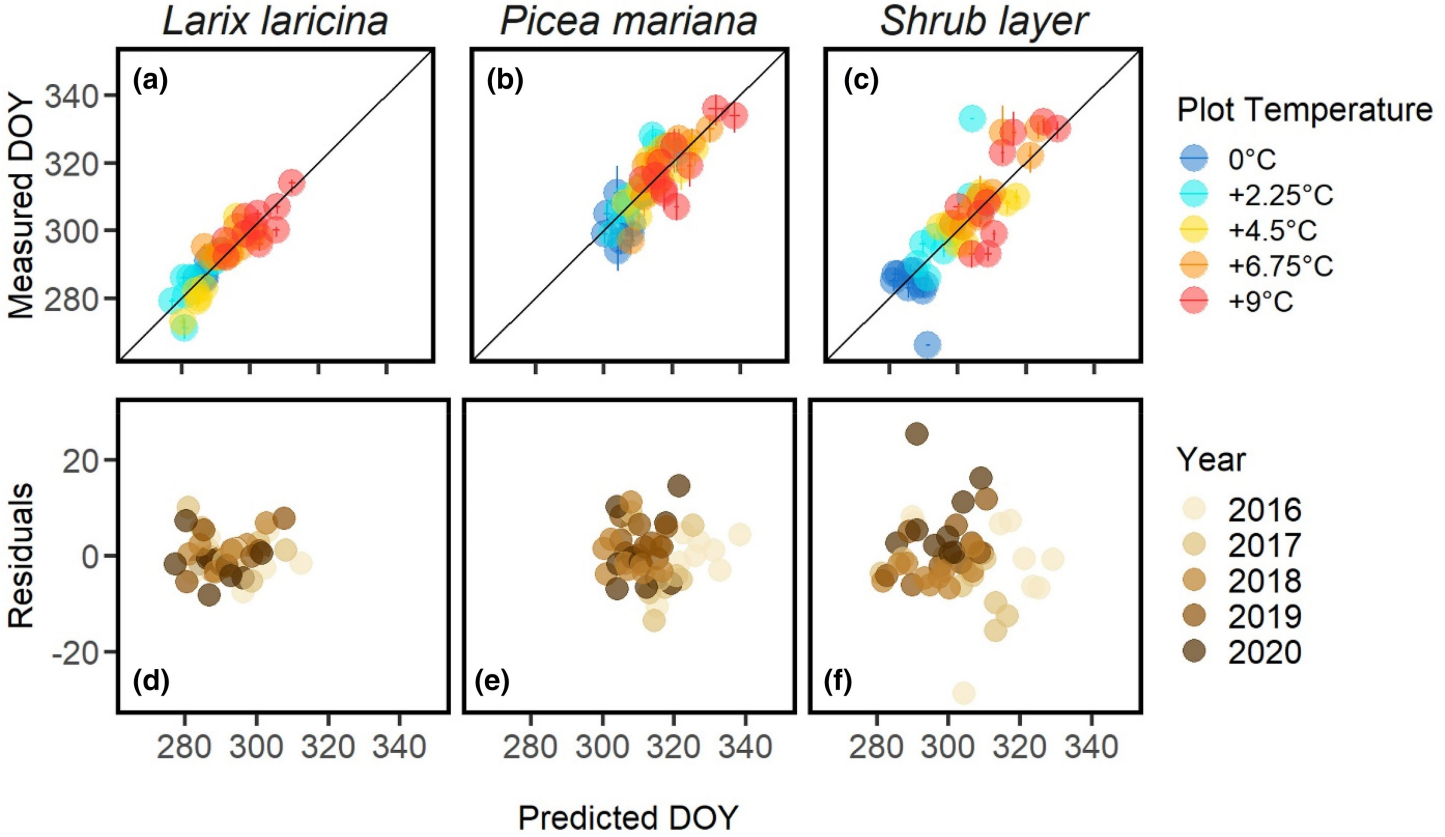
Predicted versus measured day of year (DOY) for autumn green‐down showing results from the best autumn model of the 25 parallel chains based on lowest AIC for (a) larch – CDDs_CO_2_ model, (b) spruce and shrub (c) – CDD_CO_2_ model. In (a)–(c) different colors represent targeted differential plot temperatures and in (d)–(f) different colors represent different calendar years. Air temperature was used for larch and soil temperature at 5 cm depth was used for spruce and at 0 cm depth for shrub. Variation in (a)–(c) shows upper and lower 90% confidence interval for the measured DOY transition dates and standard deviation for predicted DOY transition dates.

For all three plant functional types, including a CO_2_ parameter (Table S4) markedly improved model fits in autumn models (Figure S2, Table S3). Model residuals were symmetrical for the selected models and did not show any clear patterns (Figure 5d–f). The best model for green‐down based on lowest AIC was the CDD model with sigmoidal temperature response (CDDs_CO_2_) for *Larix*, and the default CDD model (CDD_CO_2_) for *Picea* and shrub (Figure 5, Table S3), showing the key role of cool temperatures in driving the progression of senescence. Adding water table depth to the autumn models resulted in much higher AICs providing no support for those models. The best autumn model for shrub has a CO_2_ parameter indicating that even though the linear mixed‐effects model did not show a significant CO_2_ effect, accounting for elevated CO_2_ improved model fit and provided the lowest AIC. Overall, the modeling results agree with our statistical analyses in that higher atmospheric CO_2_ concentrations accelerate senescence in each of the studied plant functional types.

### 3.4 Temperature depth profiles for modeling

In the interest of identifying important drivers and mechanisms for spring and autumn transition dates, we examined whether air or soil temperature (measured along a depth profile from 0 to −200 cm) is the better driver explaining spring green‐up and autumn green‐down. Thawing ice and increasing rooting zone temperatures in spring could provide an important signal for moisture and nutrient availability to plants and the richness of environmental data provided at SPRUCE allows this unique approach of comparing different temperature datasets. Yet for all plant functional types, using air temperature resulted in the smallest mean AIC in the best spring models (Figure S4). For autumn models, air temperature was the best dataset for *Larix* and surface temperatures of 0 and 5 cm provided best results in *Picea* and shrub (Figure S5). Generally, AICs and RMSEs increased with the depth at which soil temperature was measured; this was most pronounced in *Picea* and shrubs. Therefore, spring green‐up and autumn green‐down are most responsive to changes in air and surface soil temperatures and the high frequency variation typical of the spring and autumn transition periods.

### 3.5 Validation sites

Using the fitted parameter values derived from the best spring and autumn models from the SPRUCE experiment, we obtained decent agreement of predicted and measured transition dates when applied to PhenoCam sites with similar vegetation (Figure S3). Root mean squared errors were 9 days for *Larix* (SM1b model), 11.2 days for *Picea* (SM1 model), and 18.4 days for the shrub layer (Pab model). Surprisingly, some other models, which did not have much support in the SPRUCE data based on AIC, actually performed better in the independent validation resulting in lower RMSEs (4.2 days for *Larix* and LIN model; 7 days for *Picea* and UM1 model; 5.8 days for shrub and SGSI model), perhaps due to differences in their canopy structure or degree of exposure.

Autumn transition dates were also well predicted using the CDDP model for *Larix* and the CDD model for *Picea* and shrub (RMSE of 3 days in *Larix*, RMSE of 6.1 days in *Picea*, and RMSE of 3.3 days in shrub). This exercise showed that a small set of closely related models worked well in independent validation across all three plant function types.

## 4 DISCUSSION

The PhenoCam dataset from the SPRUCE experiment provides a unique opportunity to test how well spring and autumn models and their underlying drivers and processes predict spring green‐up and autumn green‐down when warming treatments and interannual variation in weather are combined. Daylength at the SPRUCE experiment is identical for all enclosures but mean daily temperature was experimentally manipulated spanning an air temperature differential of 0°C to +9°C compared to control plots. Over the five years considered here, spring green‐up occurred −1.1 days earlier per degree Celsius warming and autumn green‐down was delayed by 2.1 days per degree Celsius across all species (Richardson, Hufkens, et al., 2018; Schädel, Richardson, et al., 2021). Combining earlier spring green‐up and delayed autumn green‐down, the active growing season is extended by about a month in the warmest enclosures compared to ambient temperature. A larger effect of climate warming was found in autumn than spring represented by a twice as large temperature sensitivity in autumn compared to spring. This trend has previously been reported by Fu et al. (2018), who found a temperature sensitivity of 6.4 days per degree Celsius in leaf senescence of two‐year old *Fagus sylvatica* L. saplings compared to 4.5 day per degree Celsius in spring. In contrast, Menzel et al. (2006) found in a meta‐analysis a spring temperature sensitivity of 4.6 days per degree Celsius warming and an autumn temperature sensitivity of 2.4 days across a wide range of species.

In autumn, elevated CO_2_ advanced senescence in *Larix* and to a smaller extent in *Picea* but not in the shrub layer. This trend was not yet observed in Richardson, Hufkens, et al. (2018), most likely because there was no CO_2_ treatment in the first year of the experiment and data only span two years, we now added multiple years to the dataset. Direct ground observations of needle senescence in *Larix* species confirm camera‐based results although without the temperature interaction (Table S5). For other species, the ground observations did not provide evidence of a CO_2_ effect. Responses of elevated CO_2_ on autumn senescence in deciduous trees from free‐air CO_2_ enrichment experiments have been mixed (Norby, 2021). Some studies show delayed senescence (Asshoff et al., 2006; Godbold et al., 2014; Taylor et al., 2008), some show no response to elevated CO_2_ (Herrick & Thomas, 2003; Norby, Hartz‐Rubin, et al., 2003; Norby, Sholtis, et al., 2003), and a few studies show advanced senescence (Asshoff et al., 2006), especially under drought conditions that were hypothesized to reduce net carbon balance compared to ambient CO_2_ due to stomatal closure (Warren et al., 2011). A related hypothesis for advanced elevated CO_2_‐induced autumn green‐down is the carbon‐sink capacity hypothesis as described in Zani et al. (2020). The argument is that increased carbon uptake in spring and summer under elevated atmospheric CO_2_ drives earlier senescence due to late‐season growth sink limitations for assimilated carbon. Generally, it is difficult to evaluate carbon sink limitation results and multiple studies have challenged the carbon‐sink capacity hypothesis suggested by Zani et al. (Norby, 2021; e.g., Lu & Keenan, 2022).

### 4.1 Model performance

Accurately predicting spring green‐up and autumn green‐down in different species with a changing climate is important as there are large implications for the water cycle (transpiration begins when leaves emerge), the carbon cycle (plants take up carbon through photosynthesis), and land–atmosphere interactions that drive the surface water and energy balance. Plant productivity might be more influenced by an earlier spring green‐up than delayed autumn green‐down as photosynthetic capacity declines over the growing season, in some species at least (Medvigy et al., 2013). Early spring green‐up can also expose sensitive tissues to abrupt spring freeze events that could lead to net carbon losses through leave damage and delays to reach full seasonal photosynthetic activity and productivity (Gu et al., 2008; Richardson, Hufkens, et al., 2018).

### 4.2 Spring models

Based on AIC model selection, some models performed much better than others at predicting spring green‐up in response to warmer temperatures. Common to all spring models is the accumulation of thermal forcing. Adding factors such as chilling accumulation or daylength did not necessarily improve prediction of transition dates indicating that the accumulation of thermal forcing may be the single most important driver for spring green‐up. Additionally, in *Larix* the thermal degree day models (TT(s), PTT(s)) all performed well which indicates that although temperature is important there needs to be an “on switch” for plants to be sensitive to temperature. This “on switch” is represented by the starting date parameter “t0” in those models, which can also be interpreted as a photoperiod trigger.

Our study shows that even though each plant functional type had a different set of best models, there were some commonalities. Generally, sequential and parallel models performed the best and except for *Larix*, simple thermal time‐based models did not do well at all. One commonality among the sequential and parallel models are chilling requirements. Warmer winters under climate change may provide less chilling and yet the results show that chilling requirements remain a main driver under a warming climate.

### 4.3 Autumn models

Using 10 autumn models and the drivers temperature, photoperiod, and water table depth, we demonstrate that autumn green‐down in the observed species is mainly driven by declining temperature. The CDD model, which was the base model for four of the five models, progresses leaf senescence when a chilling temperature threshold is reached. Similar to the thermal time models in spring, the basic CDD model includes a fixed starting date which implies a photoperiod threshold (Basler, 2016). Previous literature has discussed the importance of accumulating CDDs (Jeong & Medvigy, 2014; Liu et al., 2015; Ren et al., 2019) and photoperiod to influence senescence (Fracheboud et al., 2009; Keskitalo et al., 2005). A meta‐analysis of northern hemisphere deciduous trees found that October temperatures were the strongest predictors of senescence followed by CDDs (Gill et al., 2015). Additionally, at higher latitudes (50° to 70° N), photoperiod exerted a strong constraint over temperature‐induced changes for autumn senescence. The authors concluded that photoperiod may play a bigger role at higher latitudes than at lower latitudes (25° to 49° N) which was confirmed by Lang et al. (2019). According to Lang et al., the SPRUCE site with a latitude of 47° N falls in the lower latitude category and photoperiod matters less than temperature.

We tested the effect of water table depth on autumn green‐down by including water table to the base CDD model. At the SPRUCE site, the warming treatment dries out surface moisture leading to a rapid decline in *Sphagnum* cover, as shown by Norby et al. (2019), likely partially due to *Sphagnum's* dependence on continual capillary wicking of water all the way to the surface. Woody species with roots anchored deeper, such as those in our study, may be less affected by some upper soil drying. Even so, in a bog setting such as SPRUCE the plants are generally shallow rooted as limited by saturated anoxic soil much of the time. As such, woody plant autumn senescence may be accelerated under strong drought conditions, which did not occur during this study. As such, the models that included water table depth did not provide a better fit in any of the plant functional types indicating that water table depth was not a driving factor of autumn senescence during this period for the species of this study.

Adding a CO_2_ parameter to each autumn model improved model fit in most cases and was particularly strong in *Larix*. By adding a CO_2_ parameter, we added an offset in the temperature forcing which allowed senescence to occur with less forcing in autumn. This provides evidence that delayed autumn senescence with warmer temperatures is counteracted by rising atmospheric CO_2_ concentrations which shortens the lengthening of the period of vegetation activity with future climate change.

### 4.4 Experimental treatment and interannual variation

The SPRUCE experiment is unique in that it provides interannual variation in weather and a very strong temperature treatment. The latter stayed constant across years while location‐specific temperature dynamics varied year by year, which together extended the range of temperature treatments by multiple degrees Celsius beyond the range of historical variability.

While none of the five experimental years included in this analysis can be categorized as extreme weather years, the warming treatment itself provides an insight to the phenological response to extreme climate. Extreme cold temperatures (as low as −15°C) naturally occur at the SPRUCE site and extreme hot temperatures are exacerbated in the warm enclosures and can exceed 45°C at 2 m above ground on some days. Extreme warm temperatures can cause drought stress on species in autumn which might counteract the extension of the growing season (Chen et al., 2020). Hot summer temperatures may not influence spring phenology but could strongly impact fall senescence, especially when coupled with dry conditions. The wide range of environmental conditions at the SPRUCE site allowed us to prove that some existing phenological models perform well with interannual climate variability.

## 5 CONCLUSION

In this study, the expectation was that using the two axes of experimental temperature treatment and interannual variability in weather, we would be able to rule out several models and identify the most important drivers and processes for spring green‐up and autumn green‐down. We find that for each plant functional type multiple models have similar RMSEs but using AIC allowed us to identify the best models. For *Picea* and shrub, the list of best spring models is short (SM1 and SMb1 for *Picea*, PAb and PA for shrub) while for *Larix*, seven models were within ∆AIC <2 (SM1b, SM1, PTT, PTTs, TT, TTs, M1). Among the best models were sequential and parallel models with chilling requirements as a common driver.

In autumn, only one model per plant functional type was identified as the best model. The accumulation of CDDs was identified as the most important driver for autumn green‐down. In addition, the best autumn models all included a CO_2_ parameter. This indicates that autumn green‐down at the SPRUCE site advances with increased atmospheric CO_2_ and that models including a CO_2_ parameter do best.

One goal of this modeling approach was to challenge boundaries of existing spring and autumn models by including interannual variability in weather and an experimental temperature range. We were able to identify spring and autumn models for each plant functional type that performed well with the environmental envelope of a warming treatment and interannual variability in weather.

## AUTHOR CONTRIBUTIONS

CS performed the analysis, interpreted the results, and wrote the manuscript. ADR contributed to the design of the study and aided in the interpretation of results. BS and KH contributed to the analysis. ADR, KJP, PJH, and JMW contributed data. All authors provided feedback on manuscript drafts and approved the manuscript for submission.

## CONFLICT OF INTEREST STATEMENT

The authors declare no conflict of interest.

## Supporting information


Data S1.

## Data Availability

The processed phenology data used for this modeling analysis are freely available under (Schädel, Richardson, et al., 2021).
