# Supplementary material for: Using long‐term data from a whole ecosystem warming experiment to identify best spring and autumn phenology models
Source: Plant Environ Interact. 2023 Jun 29;4(4):188–200. doi: 10.1002/pei3.10118 (PMC10423976; doi:10.1002/pei3.10118)
Supplement: Supplementary file 1 — Data S1. [file PEI3-4-188-s001.docx]

**Supporting Information**

**Article title:** Using long-term data from a whole ecosystem warming experiment to identify best spring and autumn phenology models

**Authors:** Christina Schädel^1,2,*,^ Bijan Seyednasrollah^3^, Paul J. Hanson^4^, Koen Hufkens^5^, Kyle J. Pearson^4^, Jeffrey M. Warren^4^, Andrew D. Richardson^1,3^

^1^ Center for Ecosystem Science and Society, Northern Arizona University, Flagstaff, AZ 86011, USA

^2^ Woodwell Climate Research Center, Falmouth, MA, USA

^3^ School of Informatics, Computing and Cyber Systems, Northern Arizona University, Flagstaff, AZ 86011, United States

^4^ Environmental Sciences Division and Climate Change Science Institute, Oak Ridge National Laboratory, Oak Ridge, TN, 37831, USA

^5^ BlueGreen Labs, 9120 Melsele, Belgium

* Corresponding author. E-mail: [cschaedel@woodwellclimate.org](mailto:cschaedel@woodwellclimate.org)

| \| LIN \| \| --- \| \| 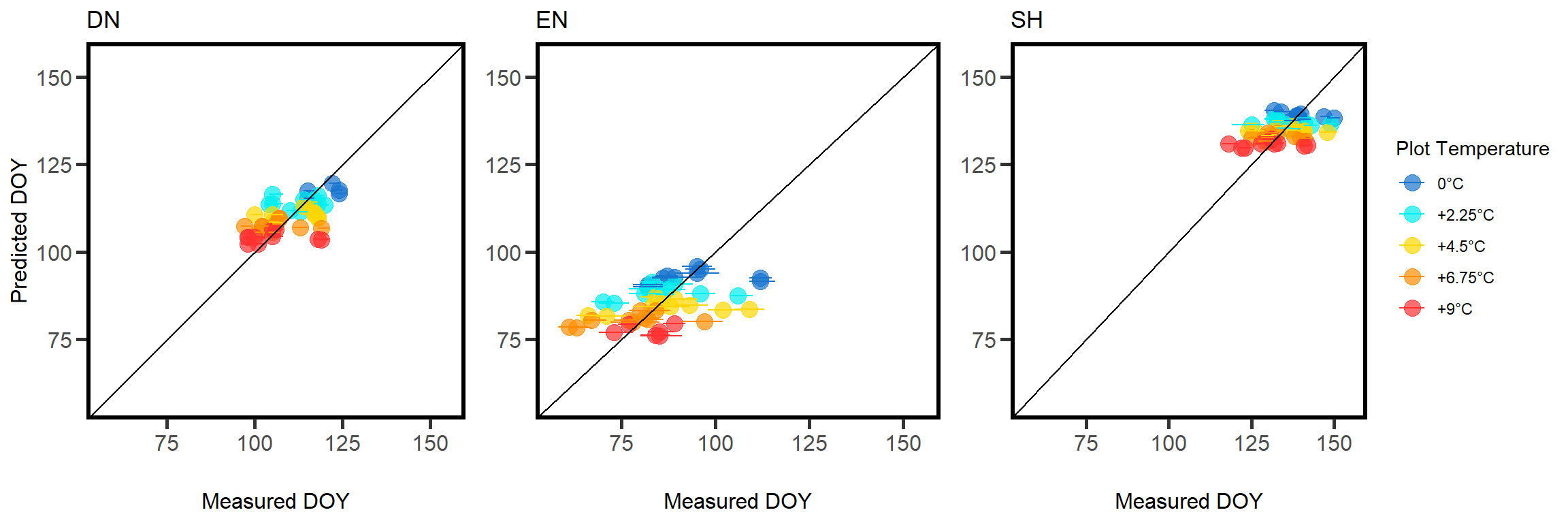 \| \| TT \| \| 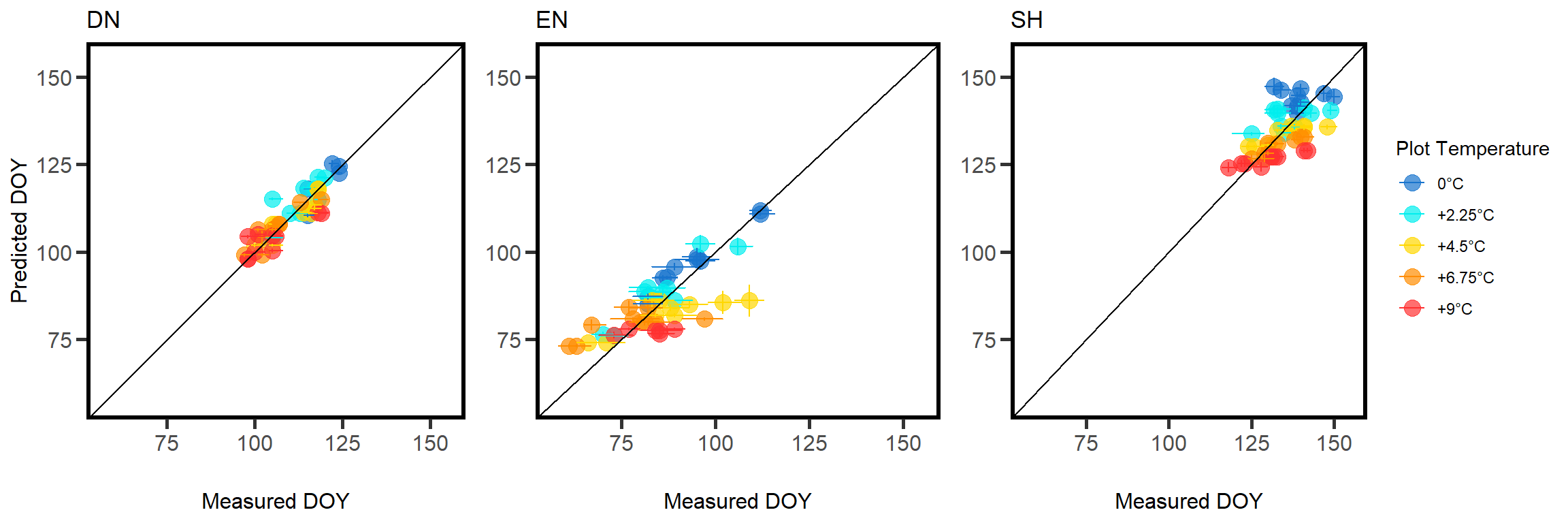 \| \| TTs \| \| 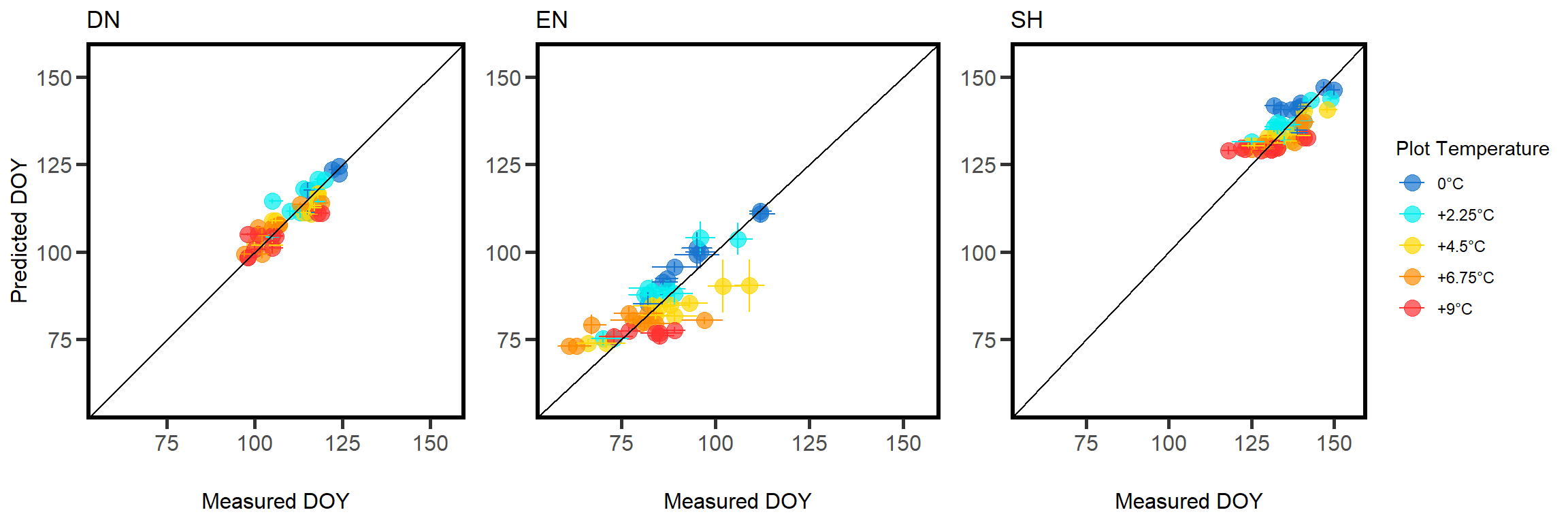 \| \| PTT \| \| 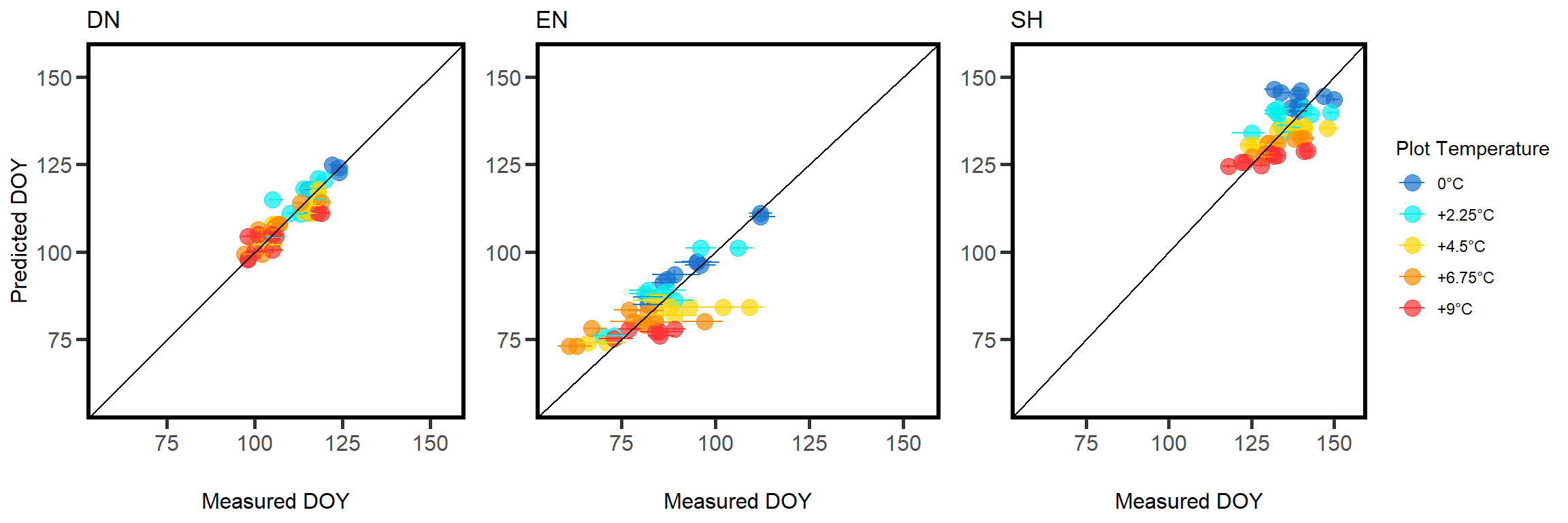 \|  \| PTTs \| \| --- \| \| 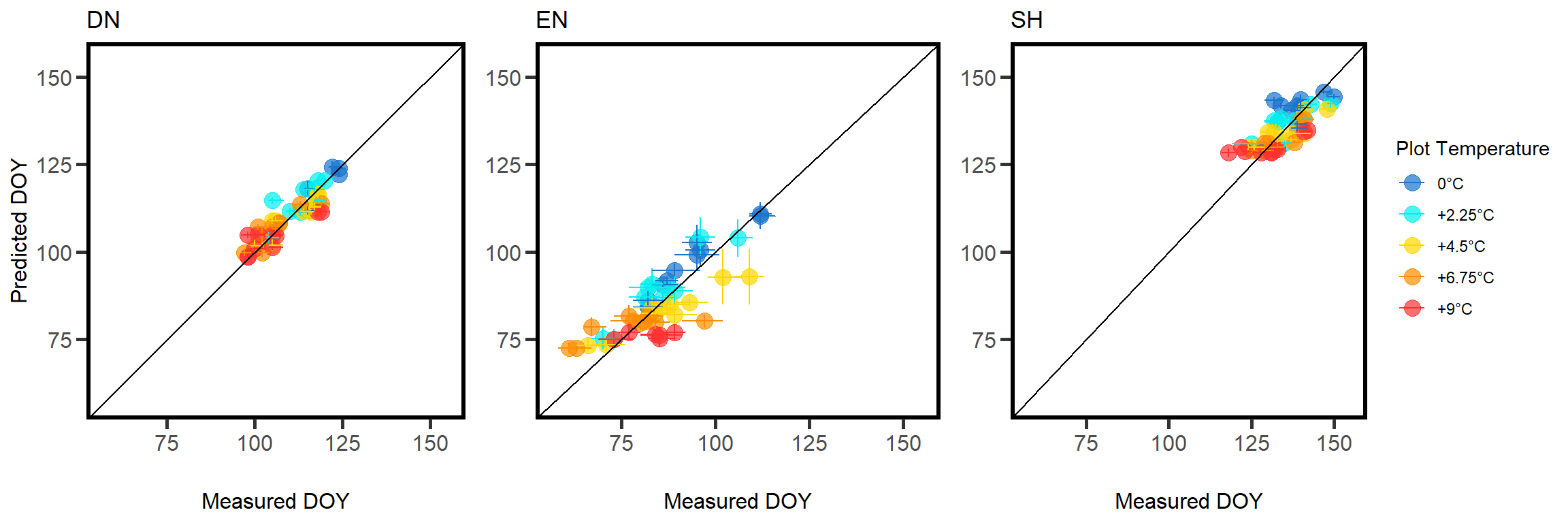 \| \| M1 \| \| 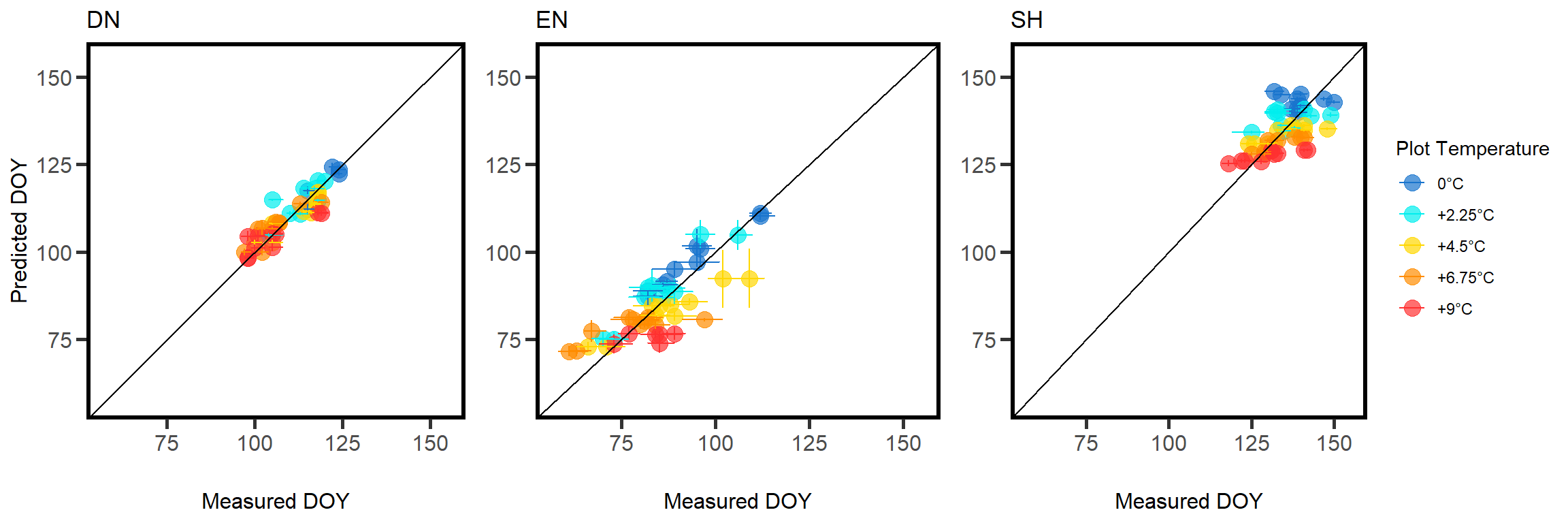 \| \| M1s \| \| 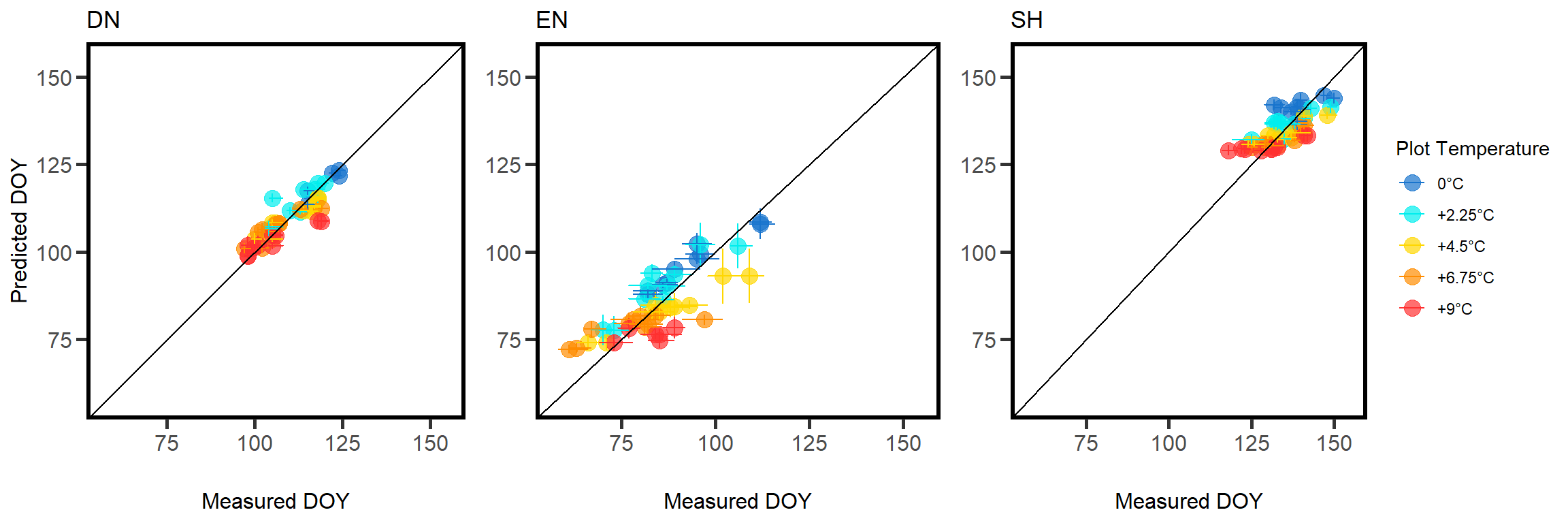 \| \| AT \| \| 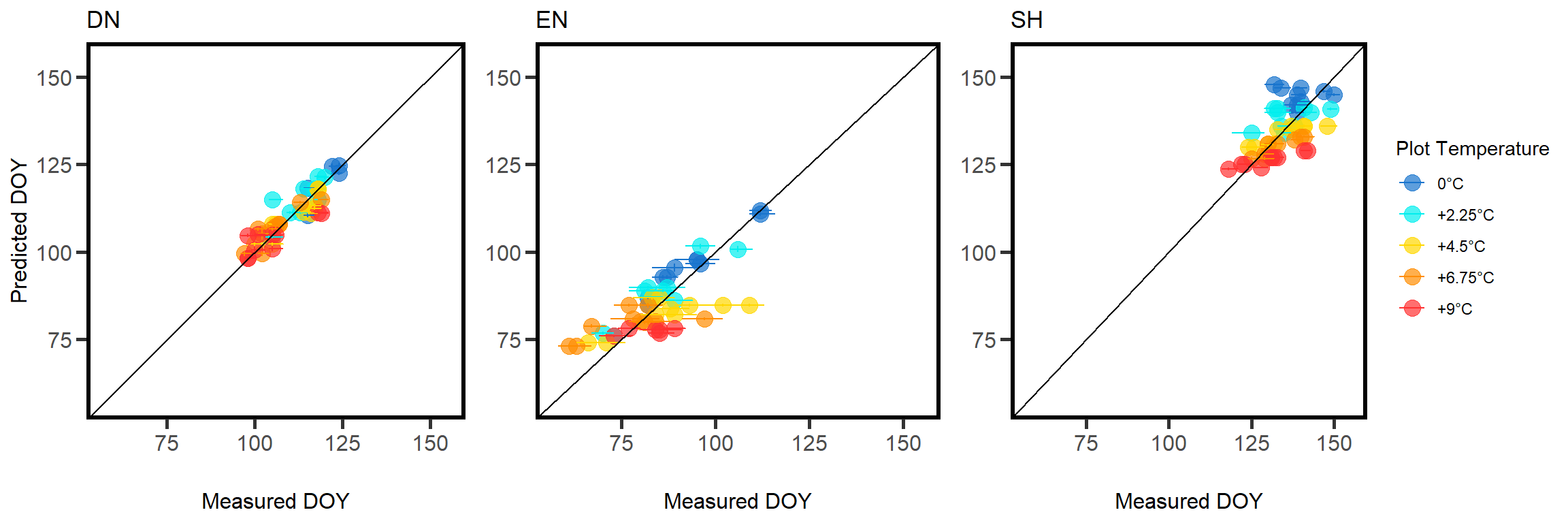 \| \|  \| \| SQ \| \| 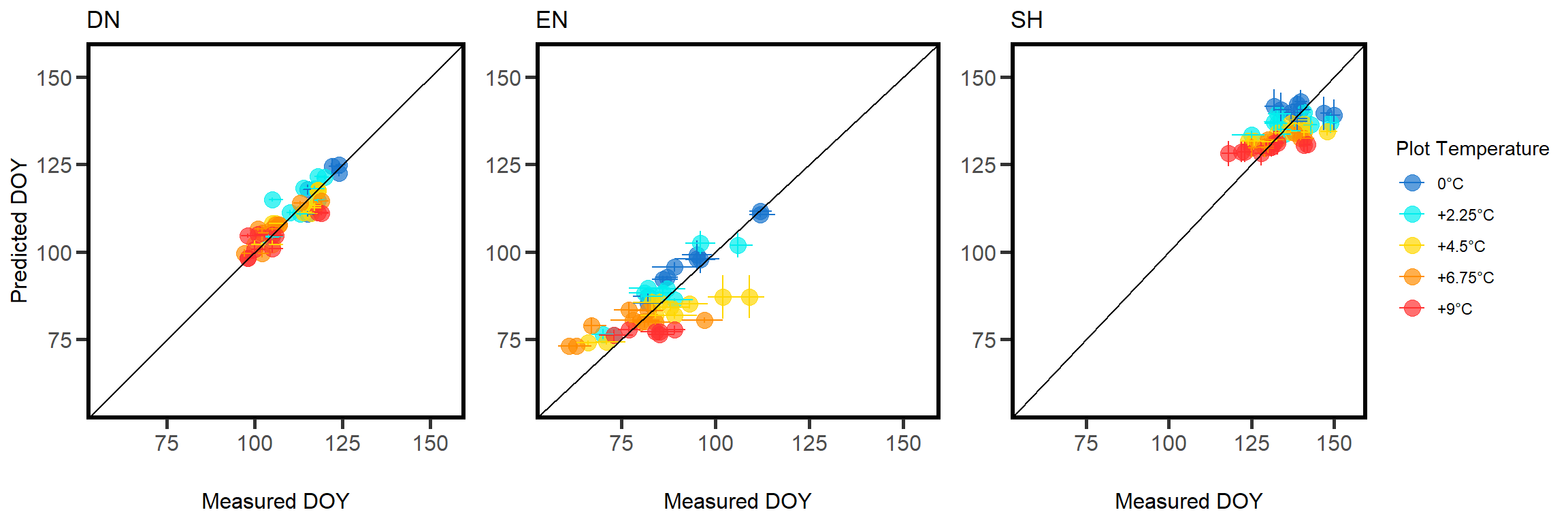 \| \| SQb \| \| 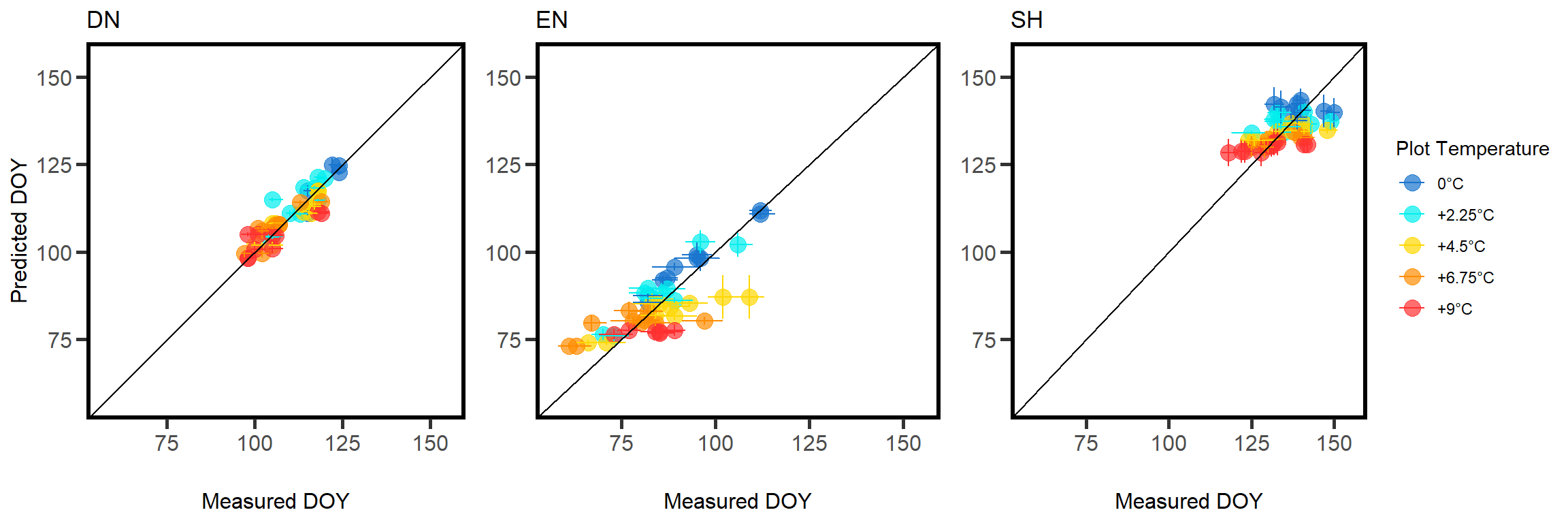 \| \| SM1 \| \| 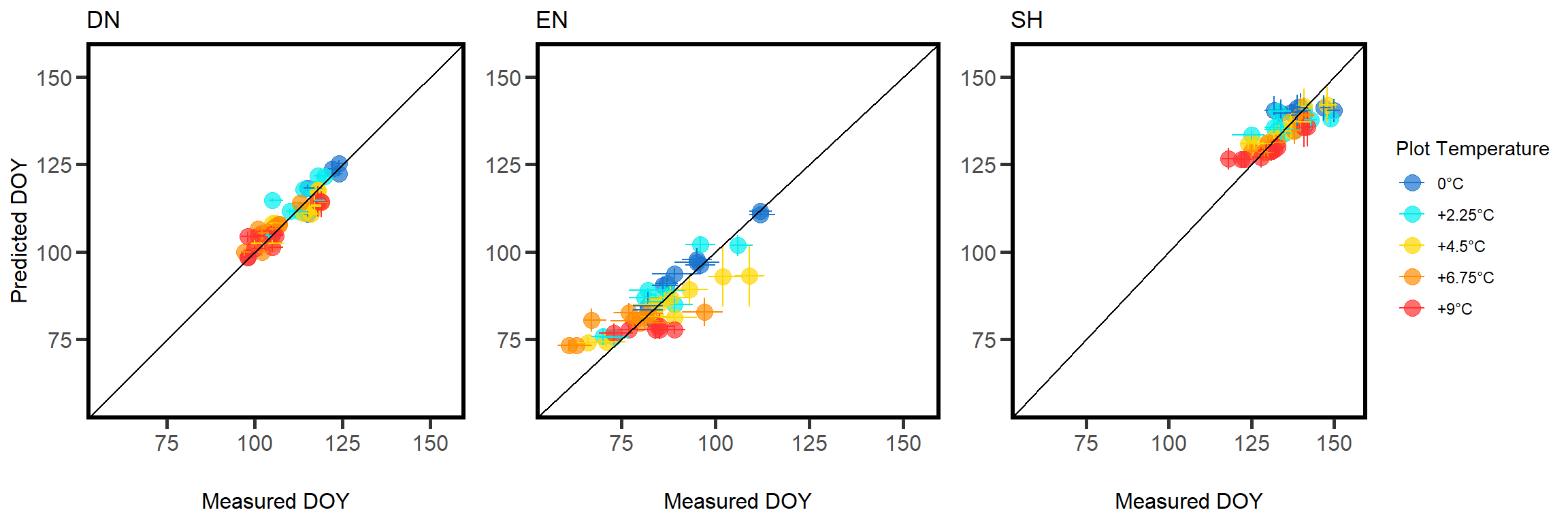 \| \| SM1b \| \| 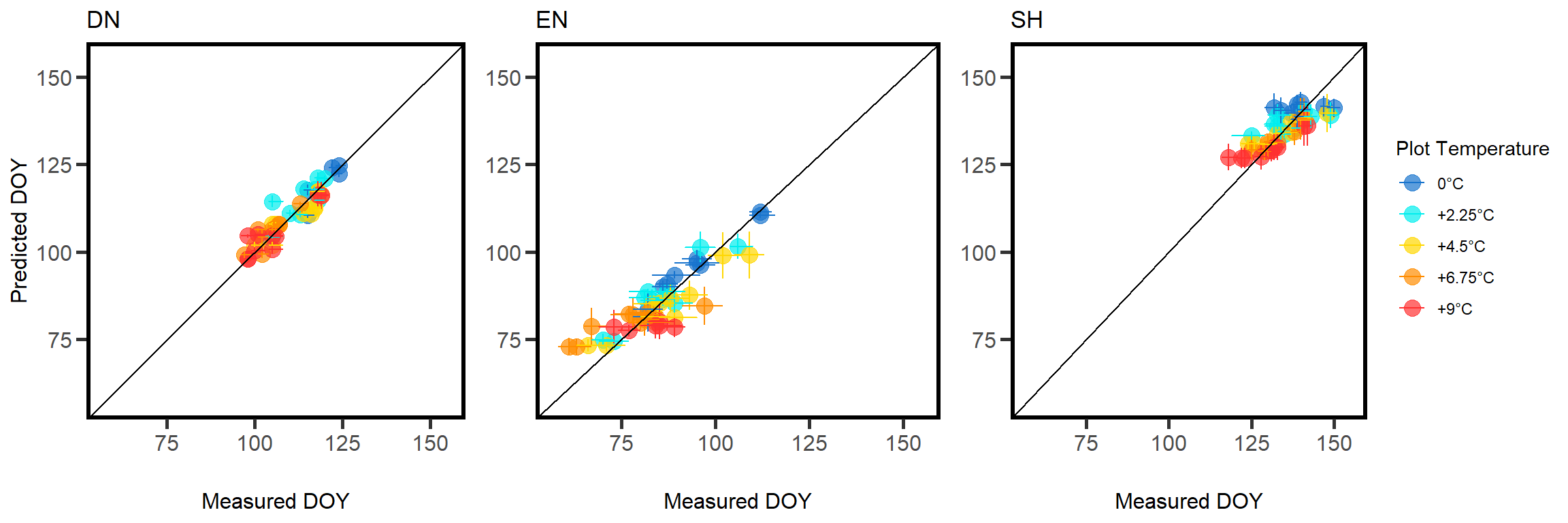 \| \|  \| \| PA \| \| 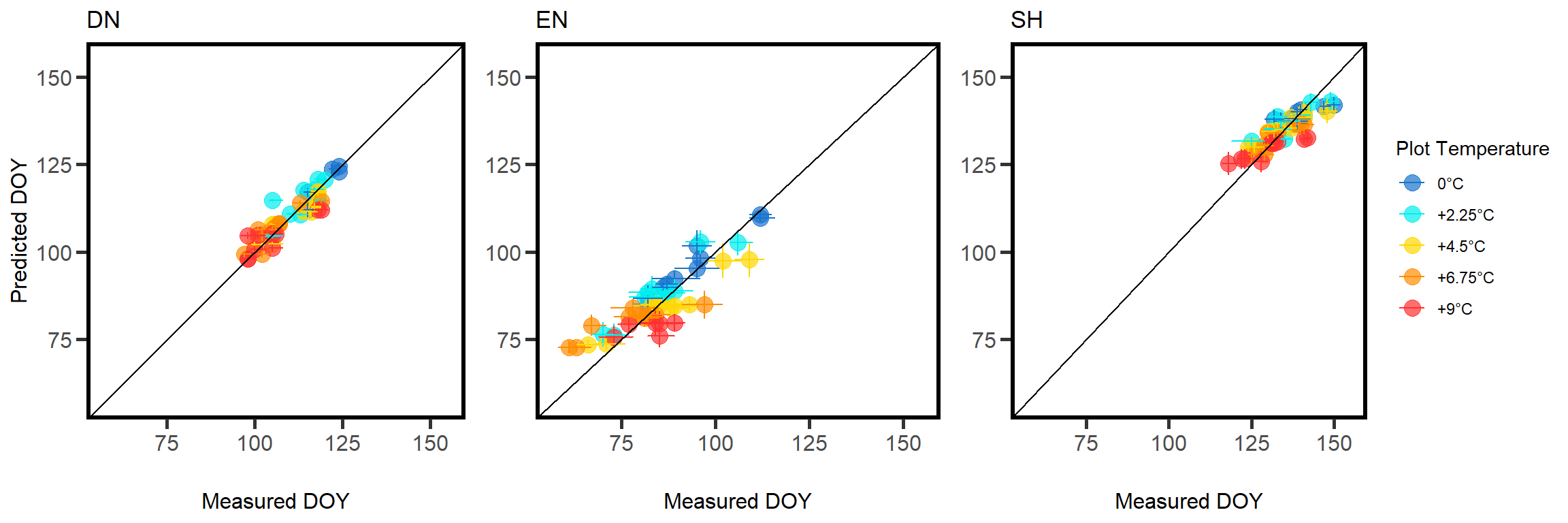 \| \| PAb \| \| 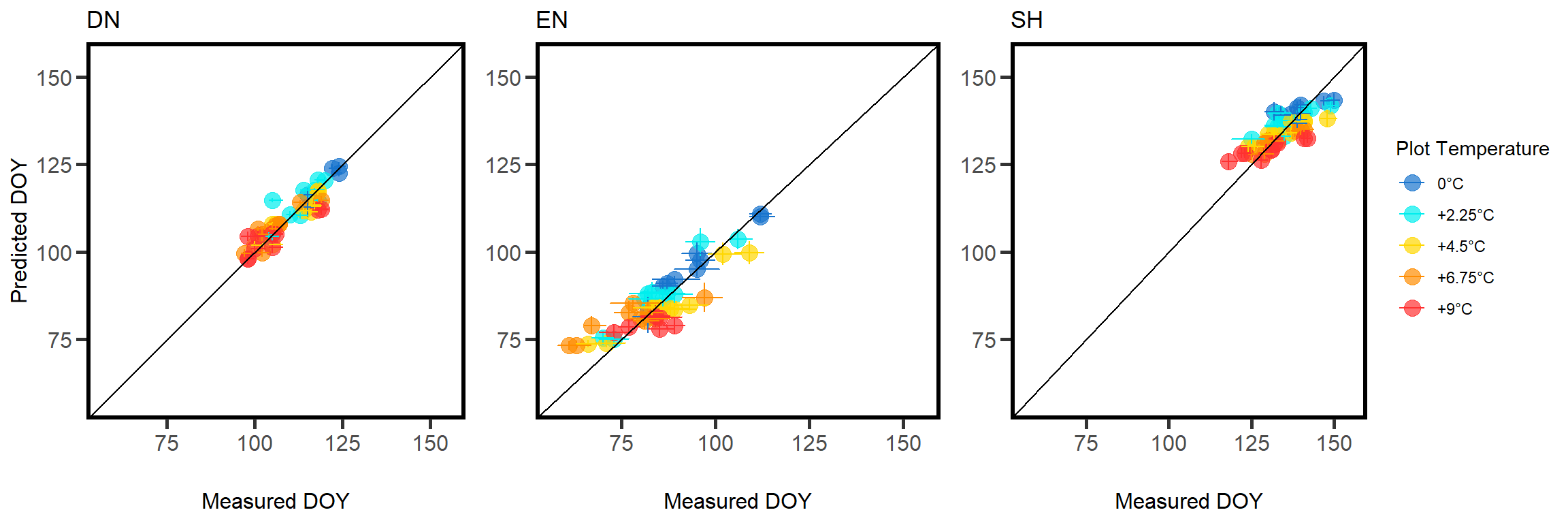 \| \| PM1 \| \| 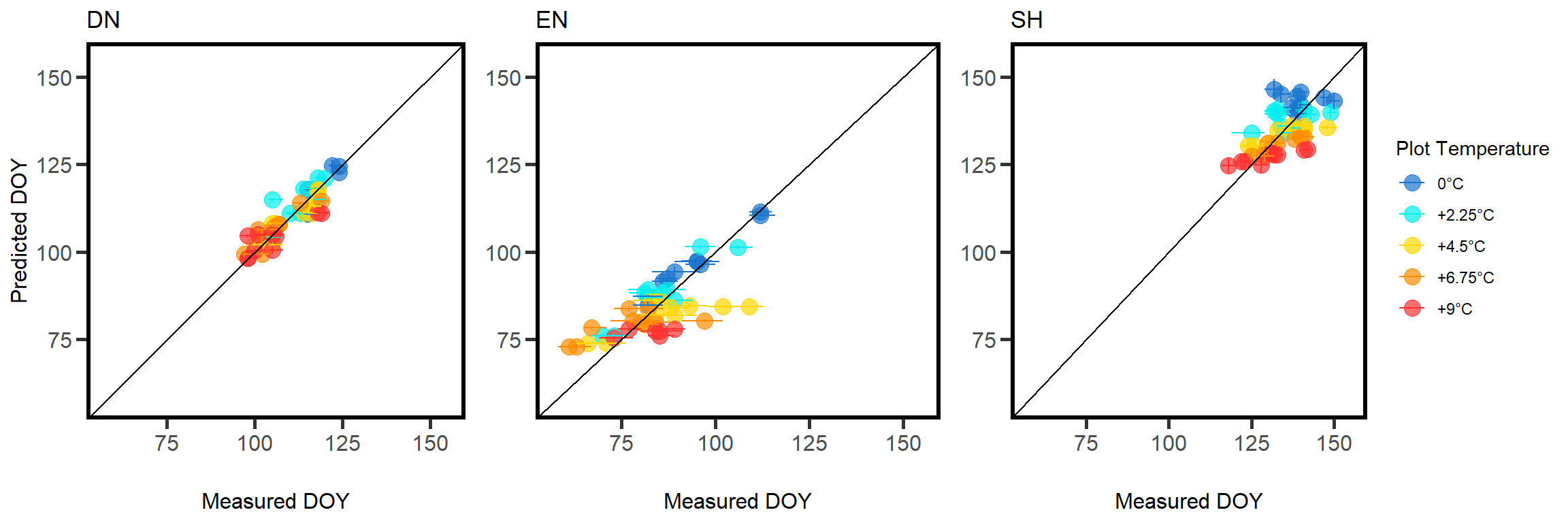 \| \| PM1b \| \| 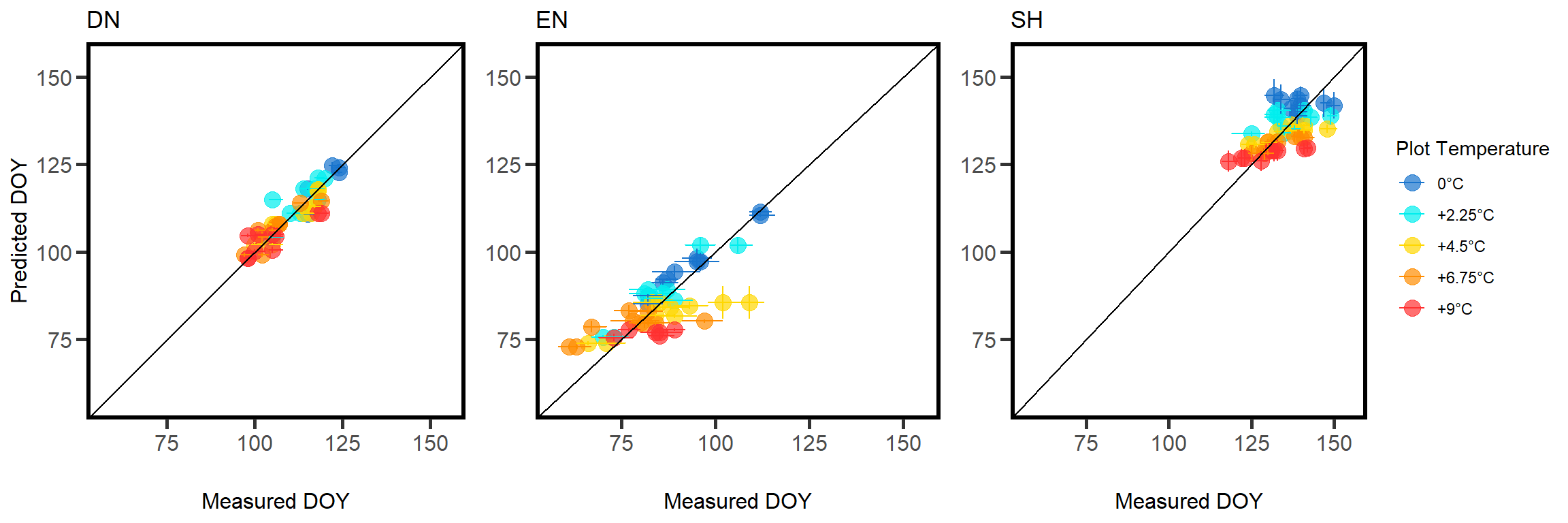 \| \| \| UM1 \| \| --- \| \| 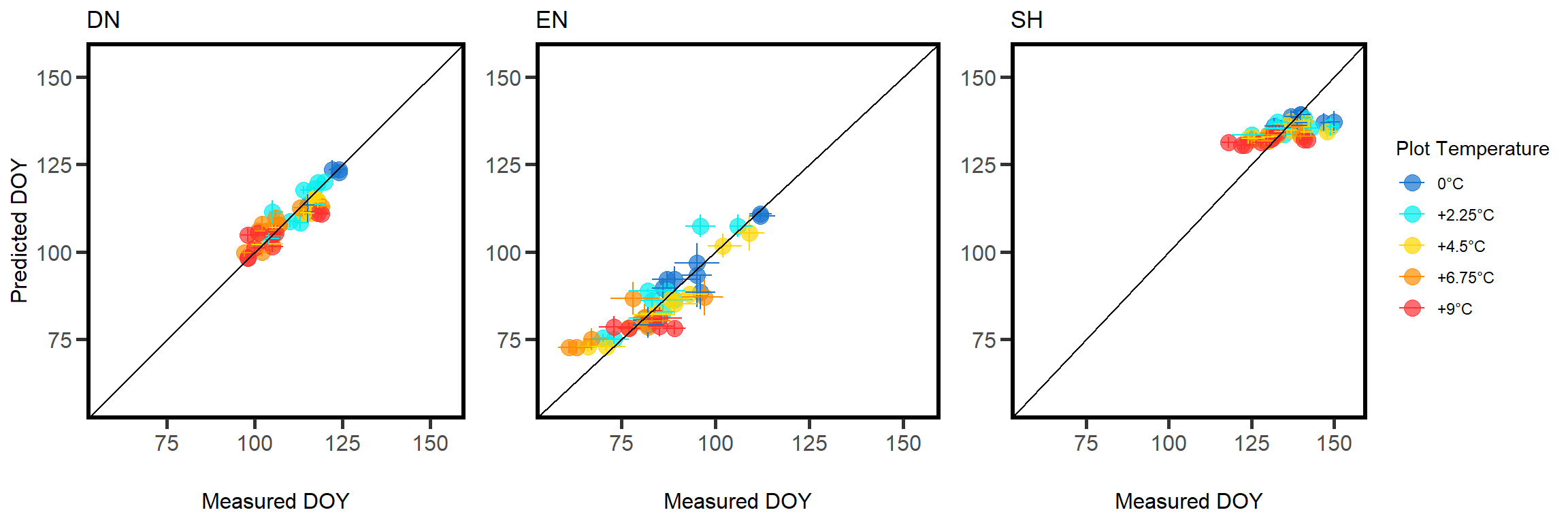 \| \| SGSI \| \| 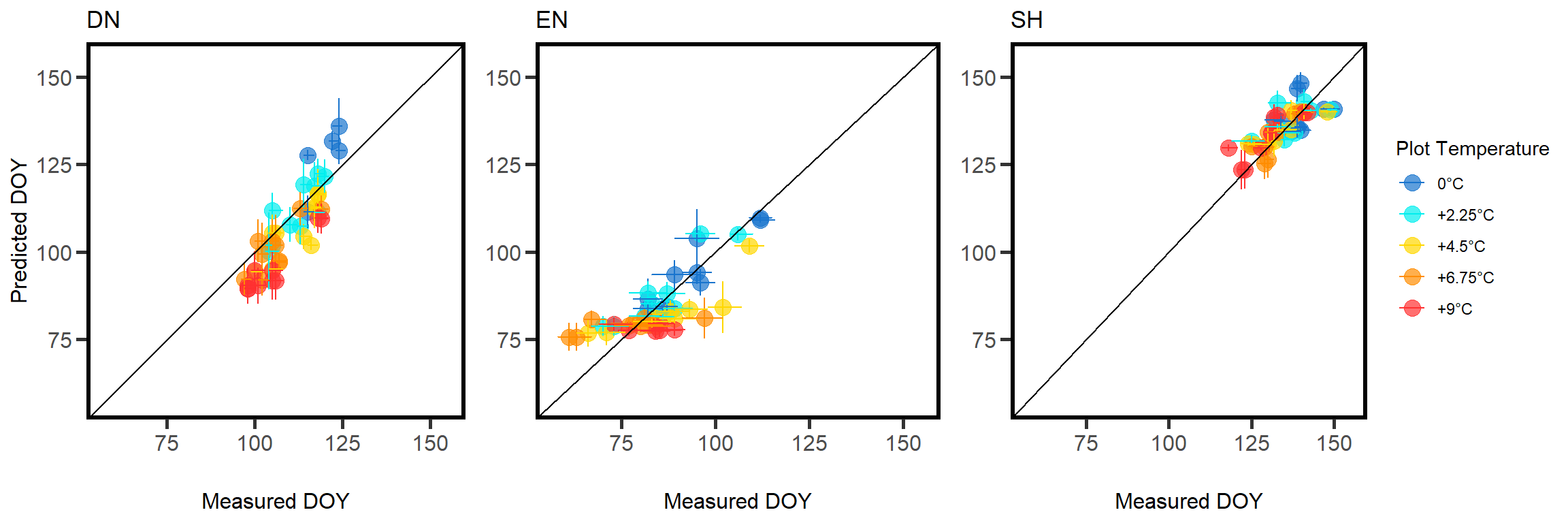 \| \| AGSI \| \| 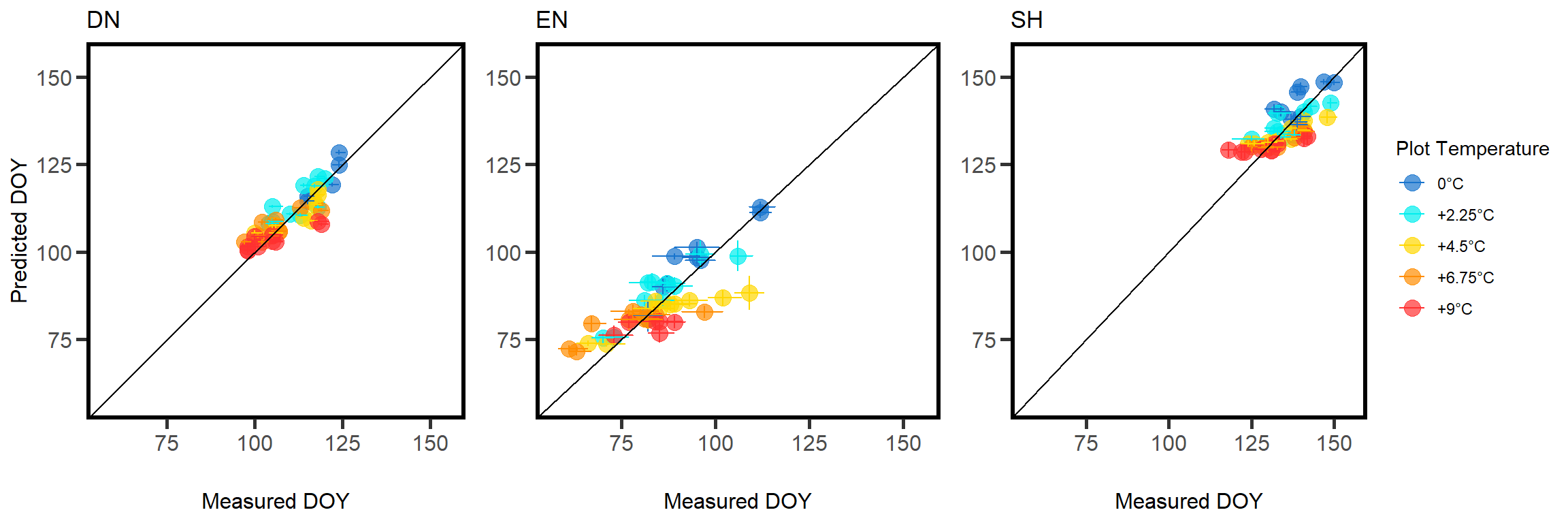 \| \|  \| \|  \| \|   **FIGURE S1** Measured versus predicted day of year (DOY) for spring green-up showing results for all 19 models as listed in Table 1 and the three plant functional types (DN=*Larix*, EN=*Picea*, SH =shrub). Different colors represent targeted differential plot temperatures. Air temperature is shown for all models. Variation shows upper and lower 90% confidence interval on the measured DOY transition dates and standard deviation for predicted DOY transition dates. |  |
| --- | --- | --- | --- | --- | --- | --- | --- | --- | --- | --- | --- | --- | --- | --- | --- | --- | --- | --- | --- | --- | --- | --- | --- | --- | --- | --- | --- | --- | --- | --- | --- | --- | --- | --- | --- | --- | --- | --- | --- | --- | --- | --- | --- | --- |
|  |  |
| CDD | |
| 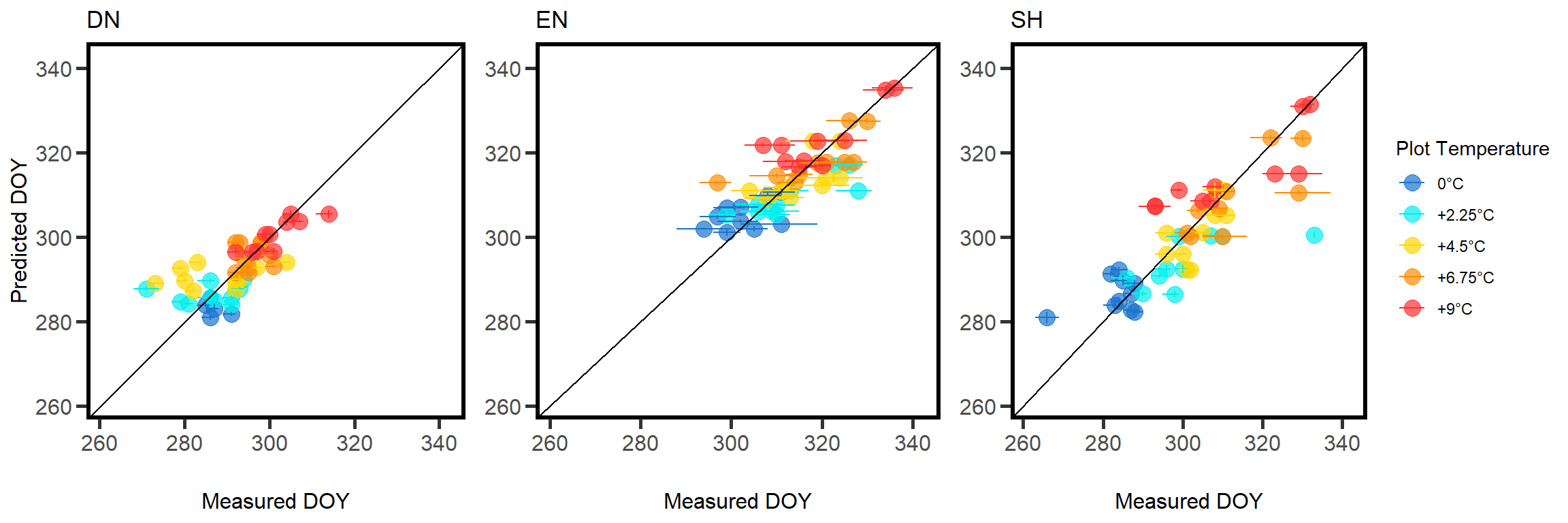 | |
| CDDCO_2_ | |
| 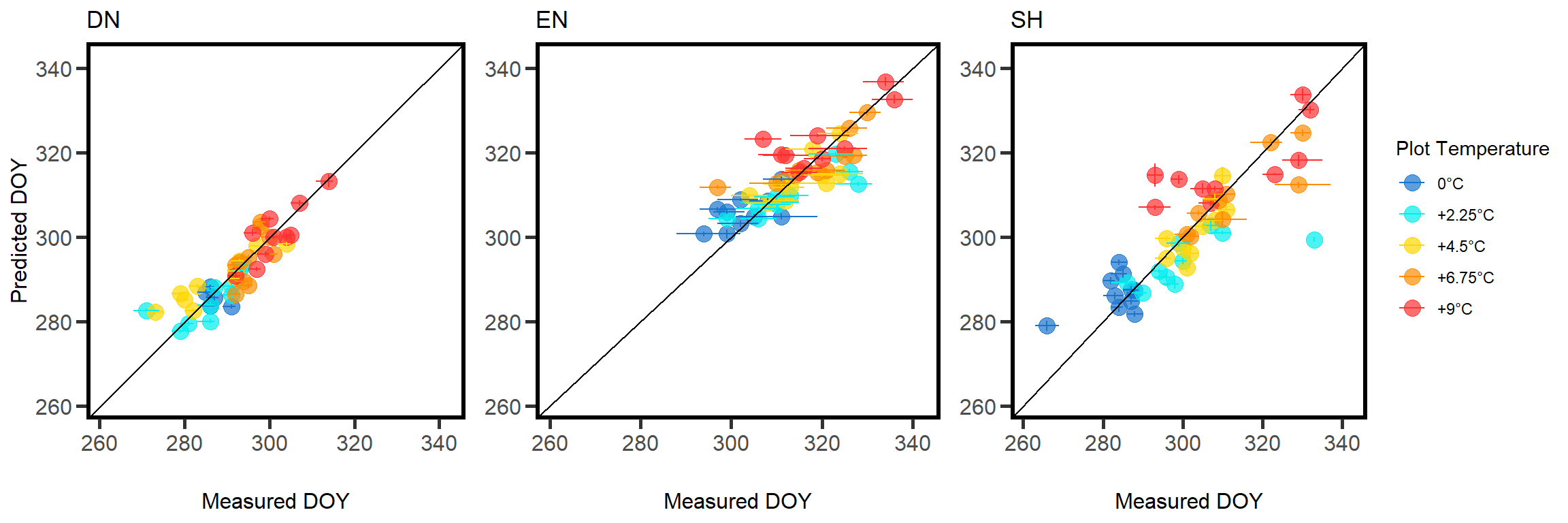 | |
| CDDs | |
| 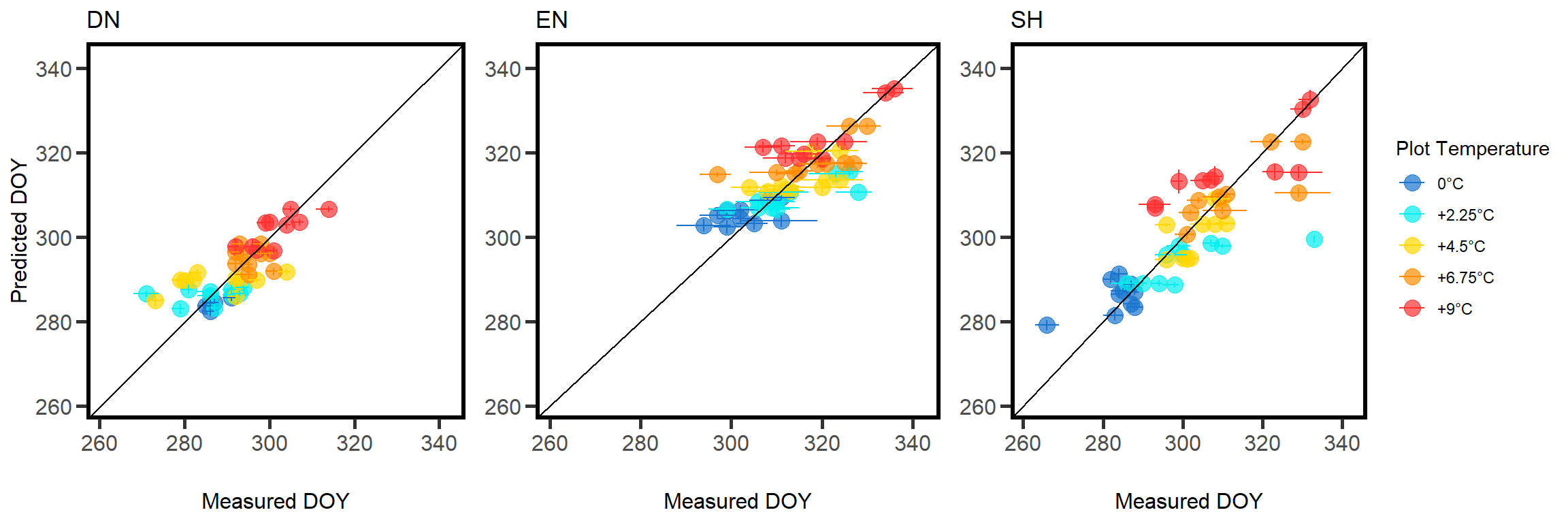 | |
| CDDsCO_2_ | |
| 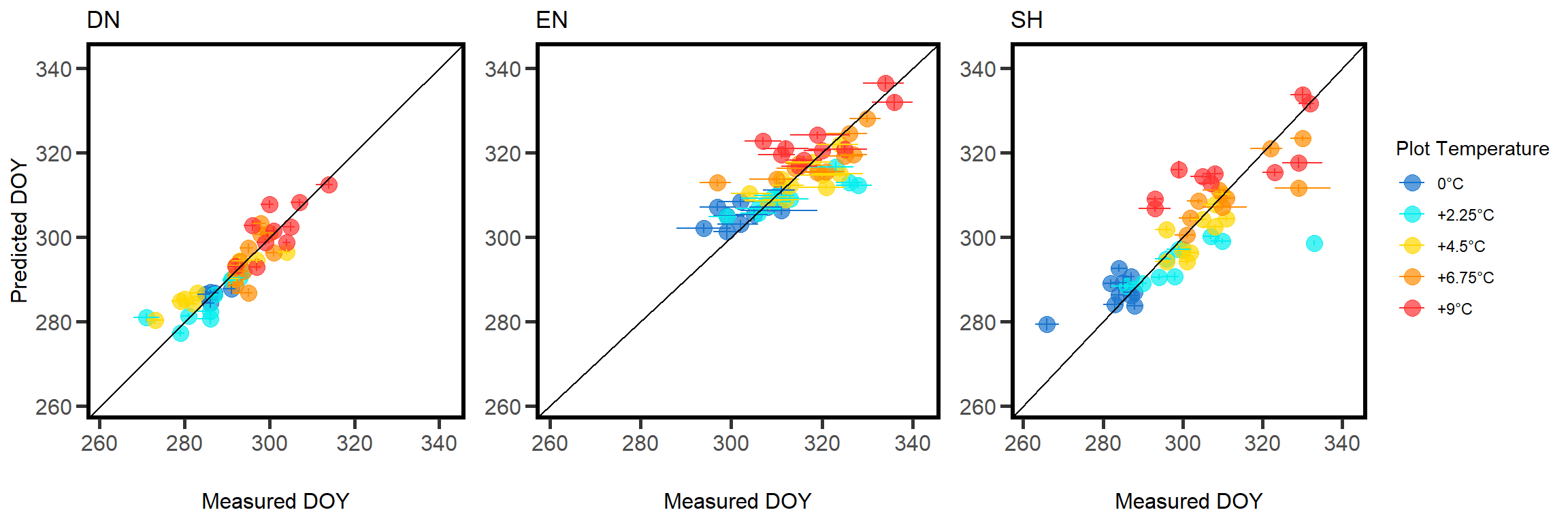 | |
| \| CDDP \| \| --- \| \| 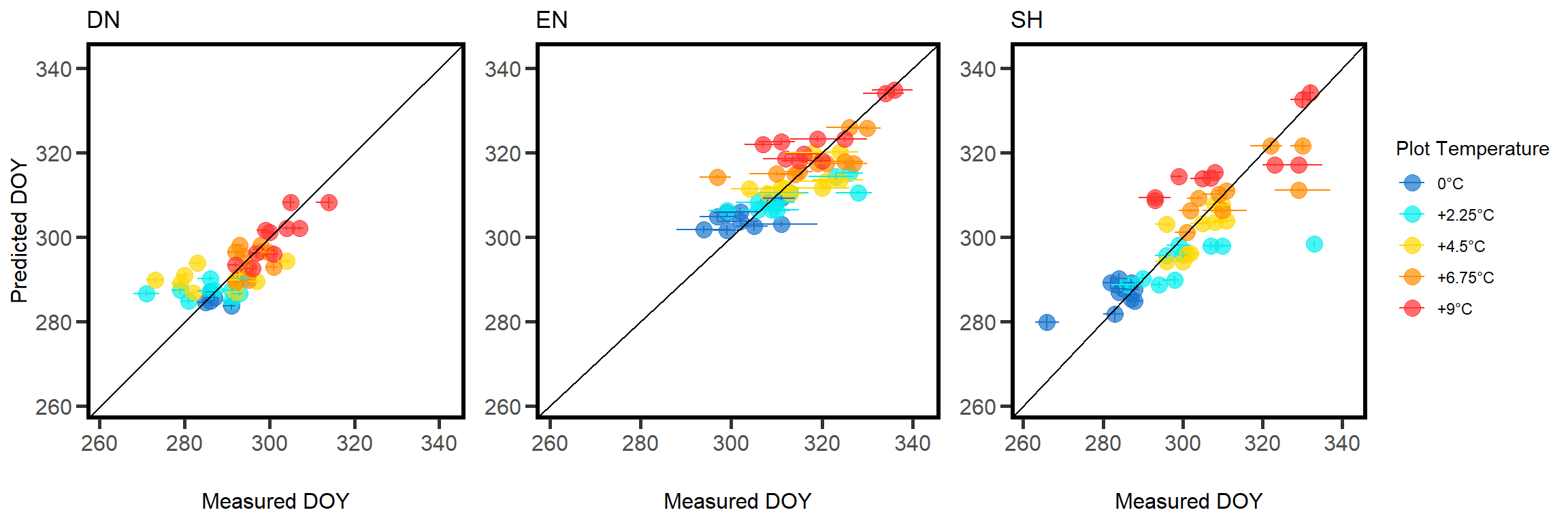 \| \| CDDPCO_2_ \| \| 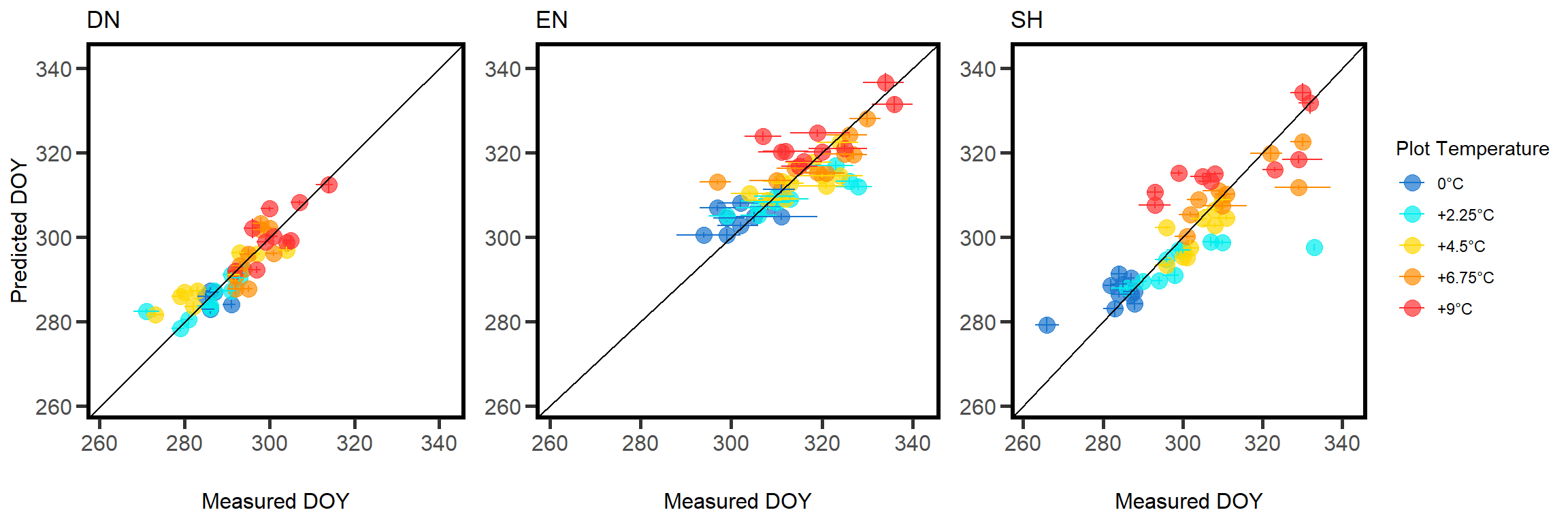 \| \| CDDM \| \| 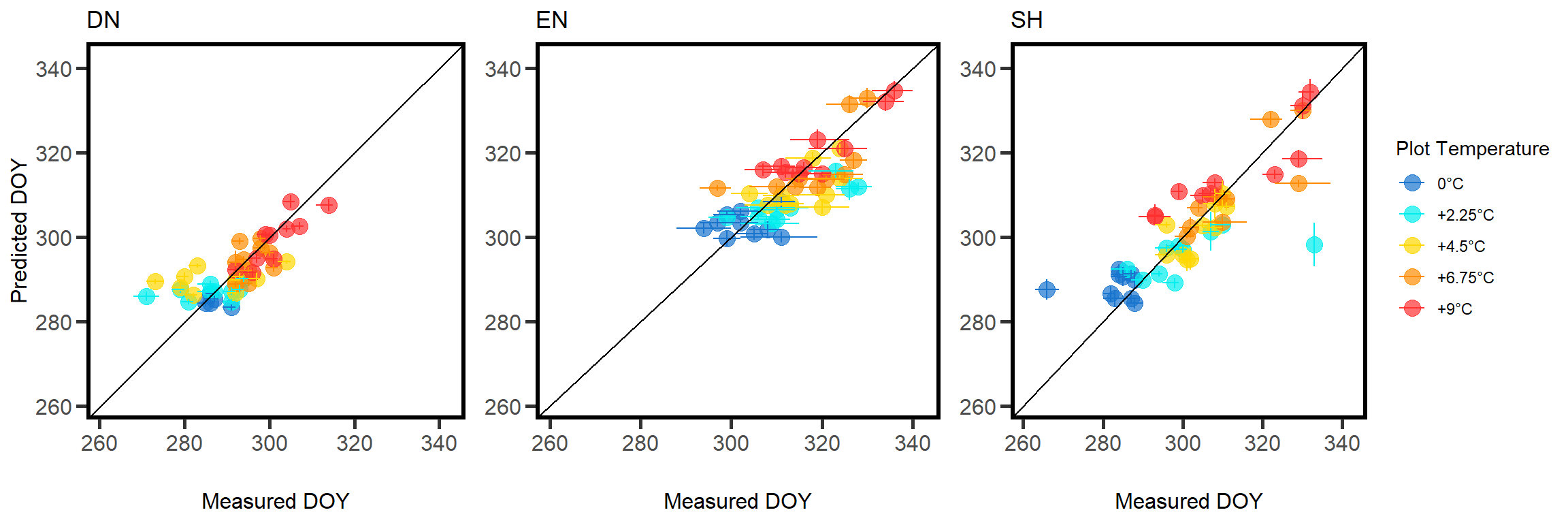 \| \| CDDMCO_2_ \| \| 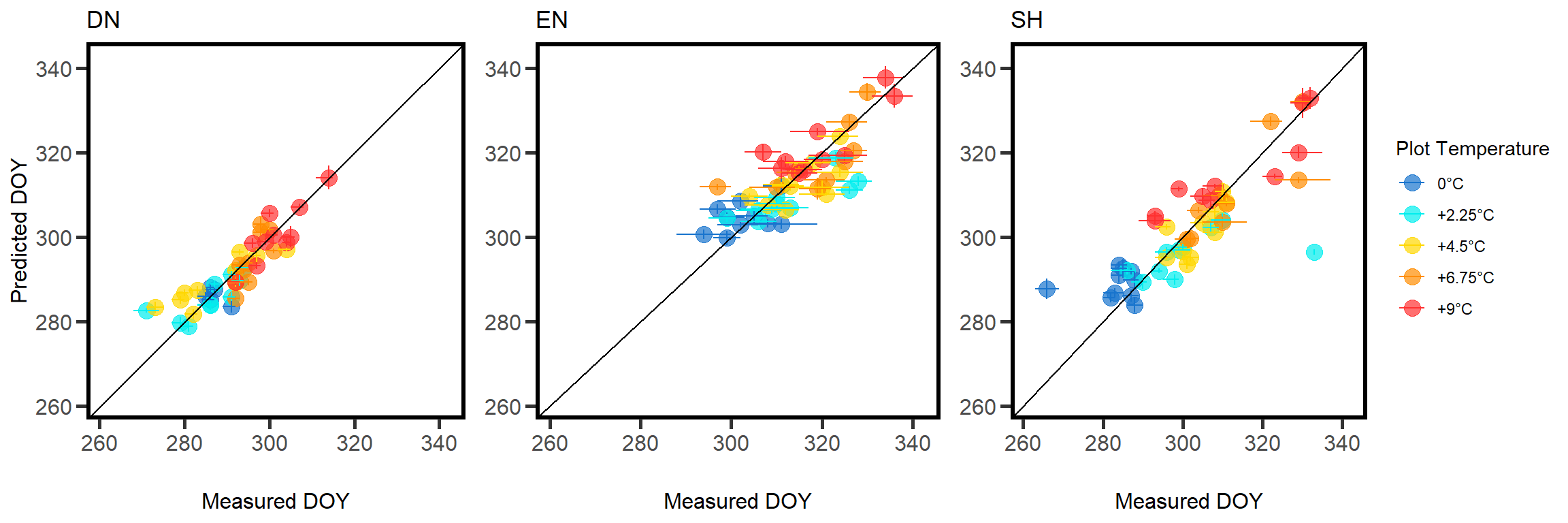 \| | |

| PPM |
| --- |
| 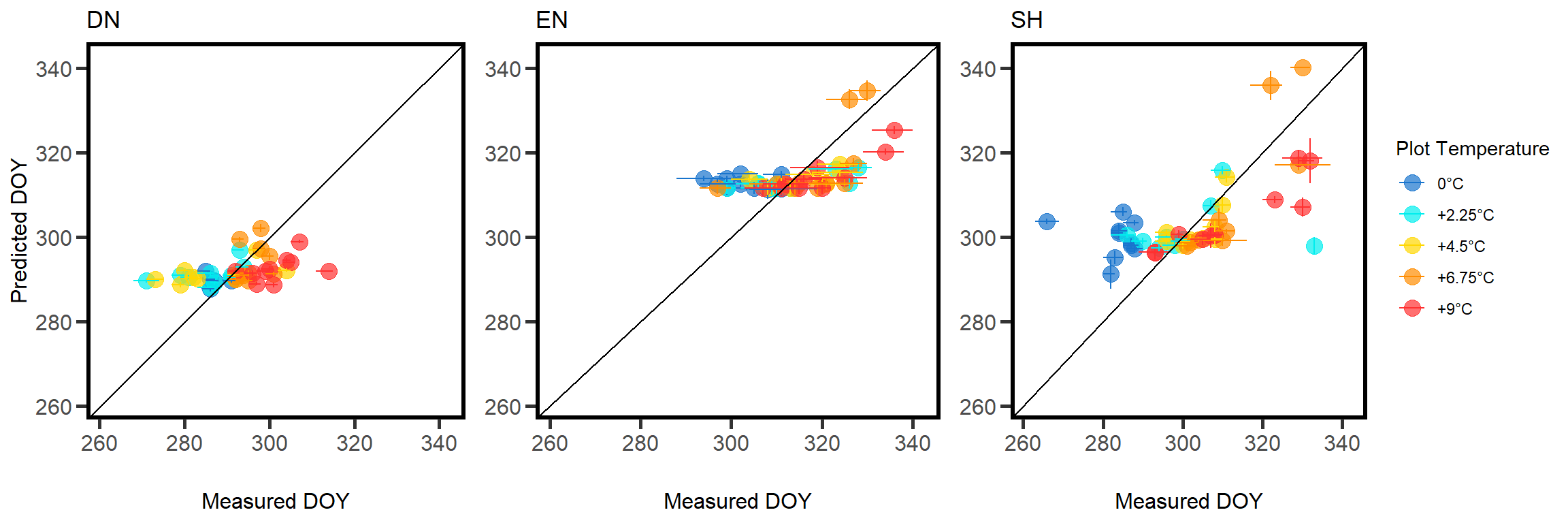 |
| PPMCO_2_ |
| 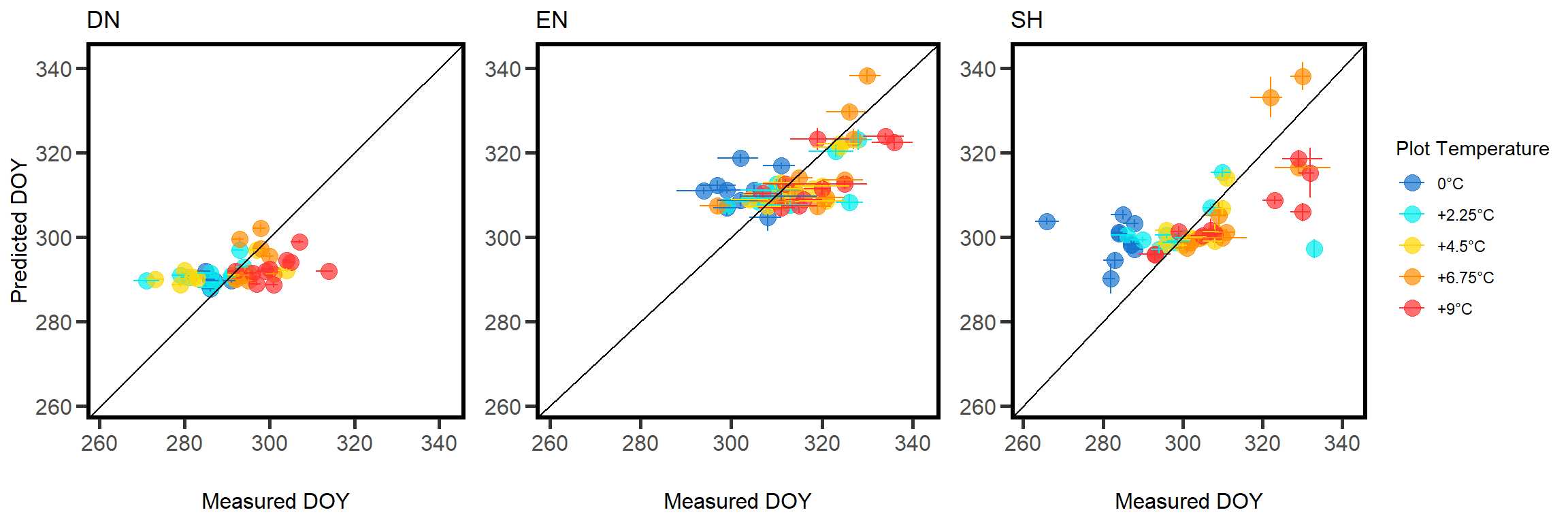 |

**FIGURE S2** Measured versus predicted day of year (DOY) for autumn green-down showing results for all 10 models as listed in Table 1 and the three plant functional types (DN=*Larix*, EN=*Picea*, SH =shrub). Different colors represent targeted differential plot temperatures. Air temperature is shown for all models. Variation shows upper and lower 90% confidence interval on the measured DOY transition dates and standard deviation for predicted DOY transition dates.

| **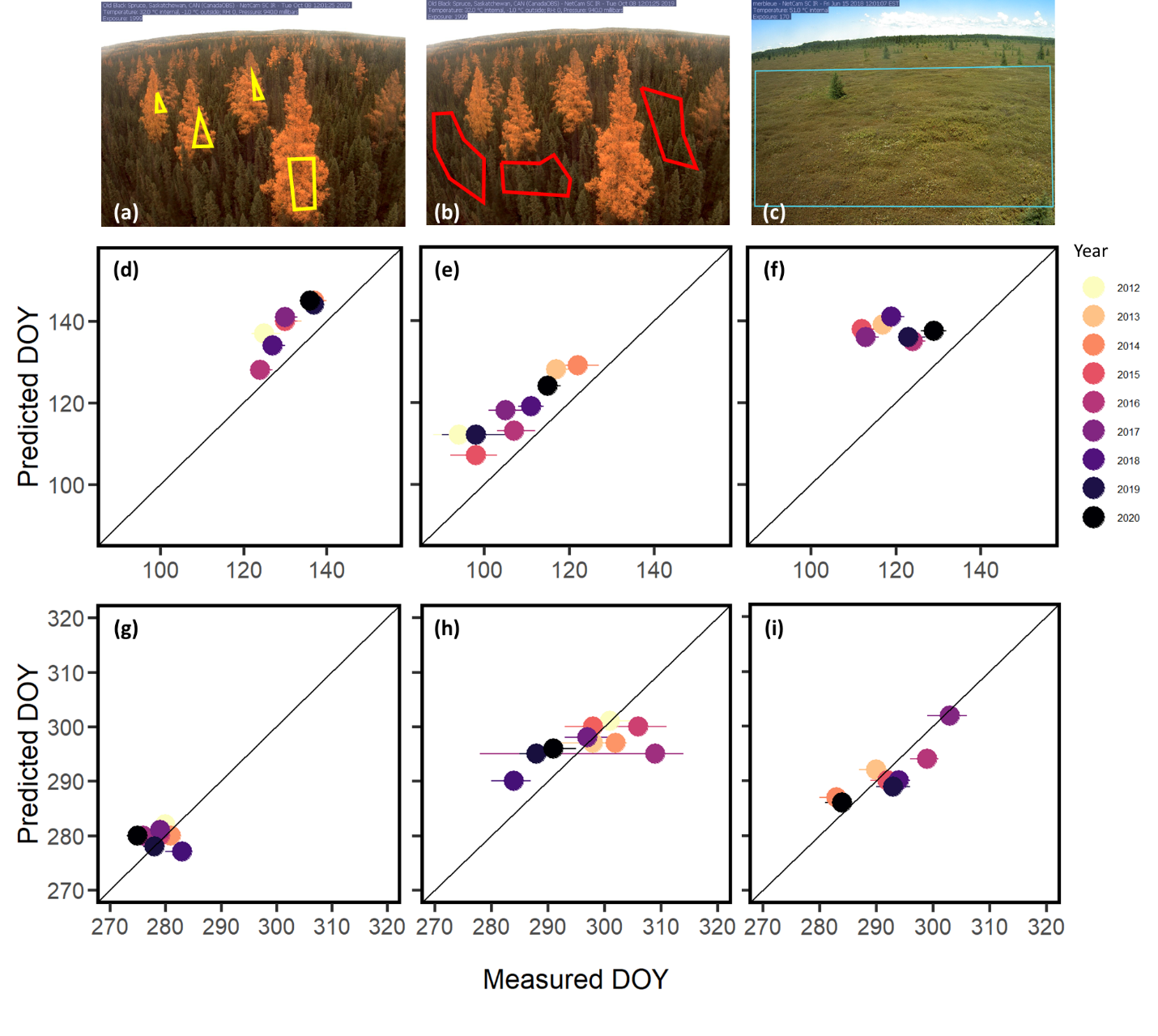** |
| --- |
| **FIGURE S3** PhenoCam image and region of interest (ROI) for the validation site larch (a) and spruce (b) at Southern Old Black Spruce Study Area (BOREAS); Prince Albert National Park, Saskatchewan, Canada. Mean annual temperature is 0.4°C, mean annual precipitation is 467mm. (c) Mer Bleue Conservation Area in Ottawa, Canada, is the validation site for the shrub layer. Measured and predicted day of year (DOY) for spring transition dates are shown for larch (d) with the SM1b model, for spruce (e) with the SM1 model, and for shrub (f) with the PAb model. In autumn, the CDDP model is shown for larch (g), and the CDD model for spruce (h) and shrub (i). Different colors represent different calendar years. Error bars shows upper and lower 90% confidence interval on the measured DOY transition dates. |

| 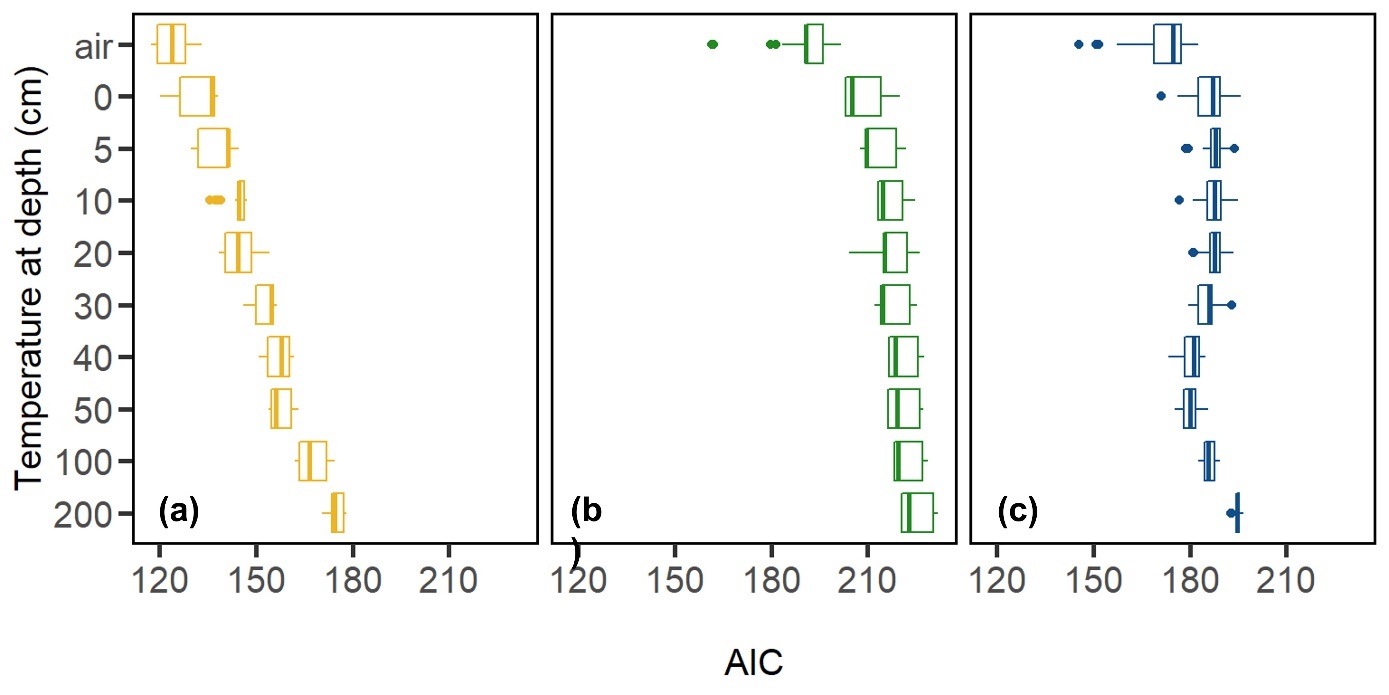 |
| --- |
| **Figure S4** AIC for temperature datasets by 2m height in air to 200 cm soil depth for the best spring model for (a) larch – SM1b, (b) spruce – SM1, and (c) shrub layer – PAb. Data are shown from 25 parallel chains. |
|  |

| 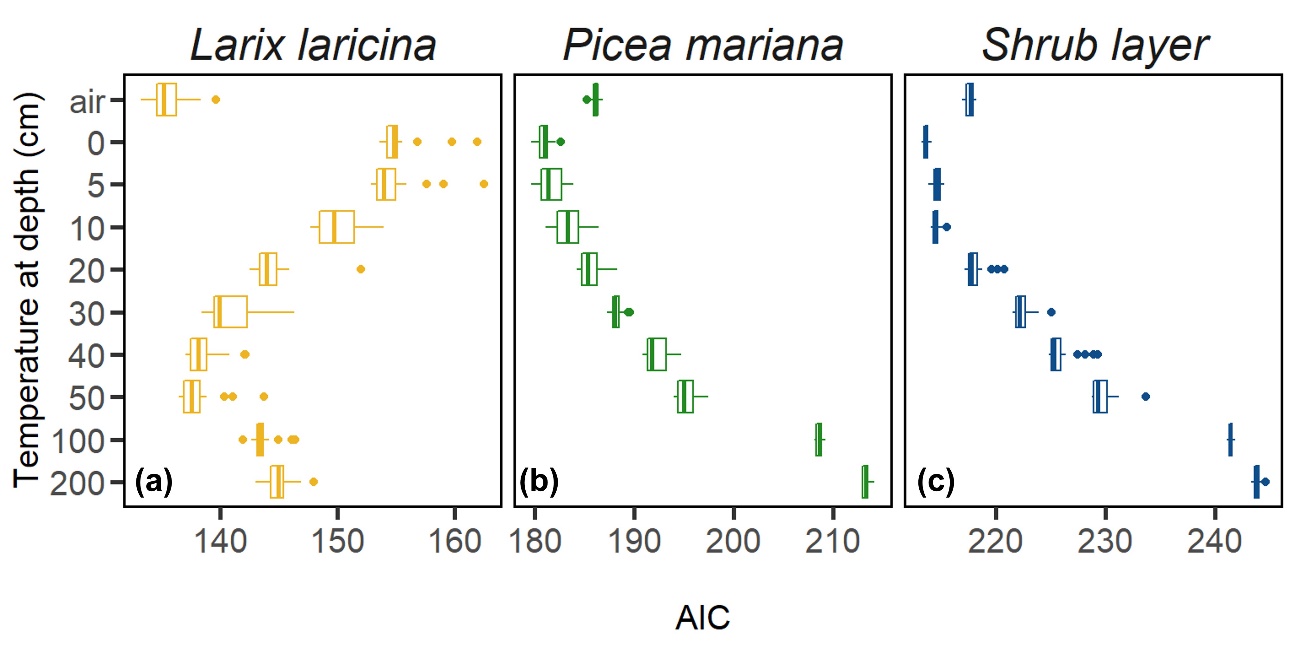 |
| --- |
| **Figure S5** AIC for temperature datasets by 2m height in air to 200 cm soil depth for the best autumn model for (a) larch – CDDs_CO_2_, (b) spruce – CDD_CO_2_, and (c) shrub layer – CDD_CO_2_. Data are shown from 25 parallel chains. Note the different x-axes. |

**TABLE S1** Lower and upper parameter limits for spring and autumn models listed in Table 1.

| **Model** | **boundary** | **t0** | **t0_chill** | **T_base** | **T_opt** | **T_min** | **T_max** | **a** | **b** | **c** | **d** | **e** | **f** | **g** | **w** | **C_ini** | **F_crit** | **C_req** | **P_crit** | **L_crit** | **D_crit** | **VPD_min** | **VPD_max** | **L_min** | **L_max** | **CO_2_** |
| --- | --- | --- | --- | --- | --- | --- | --- | --- | --- | --- | --- | --- | --- | --- | --- | --- | --- | --- | --- | --- | --- | --- | --- | --- | --- | --- |
| LIN | lower |  |  |  |  |  |  | -1000 | -1000 |  |  |  |  |  |  |  |  |  |  |  |  |  |  |  |  |  |
| LIN | upper |  |  |  |  |  |  | 1000 | 1000 |  |  |  |  |  |  |  |  |  |  |  |  |  |  |  |  |  |
| TT | lower | 1 |  | -5 |  |  |  |  |  |  |  |  |  |  |  |  | 0 |  |  |  |  |  |  |  |  |  |
| TT | upper | 365 |  | 10 |  |  |  |  |  |  |  |  |  |  |  |  | 2000 |  |  |  |  |  |  |  |  |  |
| TTs | lower | 1 |  |  |  |  |  |  | 0 | 0 |  |  |  |  |  |  | 0 |  |  |  |  |  |  |  |  |  |
| TTs | upper | 365 |  |  |  |  |  |  | 100 | 100 |  |  |  |  |  |  | 350 |  |  |  |  |  |  |  |  |  |
| PTT | lower | 1 |  | -5 |  |  |  |  |  |  |  |  |  |  |  |  | 0 |  |  |  |  |  |  |  |  |  |
| PTT | upper | 365 |  | 10 |  |  |  |  |  |  |  |  |  |  |  |  | 2000 |  |  |  |  |  |  |  |  |  |
| PTTs | lower | 1 |  |  |  |  |  |  | 0 | 0 |  |  |  |  |  |  | 0 |  |  |  |  |  |  |  |  |  |
| PTTs | upper | 365 |  |  |  |  |  |  | 100 | 100 |  |  |  |  |  |  | 350 |  |  |  |  |  |  |  |  |  |
| M1 | lower | 1 |  | -5 |  |  |  |  |  |  |  |  | 0 |  |  |  | 0 |  |  |  |  |  |  |  |  |  |
| M1 | upper | 365 |  | 10 |  |  |  |  |  |  |  |  | 5 |  |  |  | 2000 |  |  |  |  |  |  |  |  |  |
| M1s | lower | 1 |  |  |  |  |  |  | 0 | 0 |  |  | 0 |  |  |  | 0 |  |  |  |  |  |  |  |  |  |
| M1s | upper | 365 |  |  |  |  |  |  | 100 | 100 |  |  | 5 |  |  |  | 2000 |  |  |  |  |  |  |  |  |  |
| AT | lower | 1 |  | -5 |  |  |  | 0 | 0 | 0 |  |  |  |  |  |  |  |  |  |  |  |  |  |  |  |  |
| AT | upper | 365 |  | 10 |  |  |  | 500 | 1000 | 5 |  |  |  |  |  |  |  |  |  |  |  |  |  |  |  |  |
| SQ | lower | 1 | 1 | -5 | -5 | -5 | 0 |  |  |  |  |  |  |  |  |  | 0 | 0 |  |  |  |  |  |  |  |  |
| SQ | upper | 365 | 365 | 10 | 10 | 10 | 15 |  |  |  |  |  |  |  |  |  | 2000 | 350 |  |  |  |  |  |  |  |  |
| SQb | lower | 1 | 1 | -5 |  |  |  | 0 | -20 | -10 |  |  |  |  |  |  | 0 | 0 |  |  |  |  |  |  |  |  |
| SQb | upper | 365 | 365 | 10 |  |  |  | 10 | 20 | 10 |  |  |  |  |  |  | 2000 | 350 |  |  |  |  |  |  |  |  |
| SM1 | lower | 1 | 1 | -5 | -5 | -5 | 0 |  |  |  |  |  |  |  |  |  | 0 | 0 |  |  |  |  |  |  |  |  |
| SM1 | upper | 365 | 365 | 10 | 10 | 10 | 15 |  |  |  |  |  |  |  |  |  | 2000 | 350 |  |  |  |  |  |  |  |  |
| SM1b | lower | 1 | 1 | -5 |  |  |  | 0 | -20 | -10 |  |  |  |  |  |  | 0 | 0 |  |  |  |  |  |  |  |  |
| SM1b | upper | 365 | 365 | 10 |  |  |  | 10 | 20 | 10 |  |  |  |  |  |  | 2000 | 350 |  |  |  |  |  |  |  |  |
| PA | lower | 1 | 1 | -5 | -5 | -5 | 0 |  |  |  |  |  |  |  |  | 0 | 0 | 0 |  |  |  |  |  |  |  |  |
| PA | upper | 365 | 365 | 10 | 10 | 10 | 15 |  |  |  |  |  |  |  |  | 1 | 2000 | 350 |  |  |  |  |  |  |  |  |
| PAb | lower | 1 | 1 | -5 |  |  |  | 0 | -20 | -10 |  |  |  |  |  | 0 | 0 | 0 |  |  |  |  |  |  |  |  |
| PAb | upper | 365 | 365 | 10 |  |  |  | 10 | 20 | 10 |  |  |  |  |  | 1 | 2000 | 350 |  |  |  |  |  |  |  |  |
| PM1 | lower | 1 |  | -5 | -5 | -5 | 0 |  |  |  |  |  |  |  |  | 0 | 0 | 0 |  |  |  |  |  |  |  |  |
| **Model** | **boundary** | **t0** | **t0_chill** | **T_base** | **T_opt** | **T_min** | **T_max** | **a** | **b** | **c** | **d** | **e** | **f** | **g** | **w** | **C_ini** | **F_crit** | **C_req** | **P_crit** | **L_crit** | **D_crit** | **VPD_min** | **VPD_max** | **L_min** | **L_max** | **CO_2_** |
| PM1 | upper | 365 |  | 10 | 10 | 10 | 15 |  |  |  |  |  |  |  |  | 1 | 2000 | 350 |  |  |  |  |  |  |  |  |
| PM1b | lower | 1 |  | -5 |  |  |  | 0 | -20 | -10 |  |  |  |  |  | 0 | 0 | 0 |  |  |  |  |  |  |  |  |
| PM1b | upper | 365 |  | 10 |  |  |  | 10 | 20 | 10 |  |  |  |  |  | 1 | 2000 | 350 |  |  |  |  |  |  |  |  |
| UM1 | lower | 1 |  | -5 | -5 | -5 | 0 |  |  |  |  |  | -100 |  | 0 |  |  | 0 |  |  |  |  |  |  |  |  |
| UM1 | upper | 365 |  | 10 | 10 | 10 | 15 |  |  |  |  |  | 0 |  | 1000 |  |  | 350 |  |  |  |  |  |  |  |  |
| SGSI | lower |  |  |  |  | -15 | 0 |  |  |  |  |  |  |  |  |  | 0 |  |  |  |  | 0 | 2000 | 8 | 10 |  |
| SGSI | upper |  |  |  |  | 0 | 45 |  |  |  |  |  |  |  |  |  | 1 |  |  |  |  | 2000 | 5000 | 11.5 | 12 |  |
| AGSI | lower |  |  |  |  | -15 | 0 |  |  |  |  |  |  |  |  |  | 0 |  |  |  |  | 0 | 2000 | 8 | 10 |  |
| AGSI | upper |  |  |  |  | 0 | 45 |  |  |  |  |  |  |  |  |  | 365 |  |  |  |  | 2000 | 5000 | 11.5 | 12 |  |
| CDD | lower | 180 |  | -25 |  |  |  |  |  |  |  |  |  |  |  |  | -1000 |  |  |  |  |  |  |  |  |  |
| CDD | upper | 365 |  | 45 |  |  |  |  |  |  |  |  |  |  |  |  | 0 |  |  |  |  |  |  |  |  |  |
| CDD_CO_2_ | lower | 180 |  | -25 |  |  |  |  |  |  |  |  |  |  |  |  | -1000 |  |  |  |  |  |  |  |  | -250 |
| CDD_CO_2_ | upper | 365 |  | 45 |  |  |  |  |  |  |  |  |  |  |  |  | 0 |  |  |  |  |  |  |  |  | 250 |
| CDDs | lower | 180 |  |  |  |  |  |  | -2 | 0 |  |  |  |  |  |  | 0 |  |  |  |  |  |  |  |  |  |
| CDDs | upper | 365 |  |  |  |  |  |  | 0 | 30 |  |  |  |  |  |  | 50 |  |  |  |  |  |  |  |  |  |
| CDDs_CO_2_ | lower | 180 |  |  |  |  |  |  | -2 | 0 |  |  |  |  |  |  | 0 |  |  |  |  |  |  |  |  | -10 |
| CDDs_CO_2_ | upper | 365 |  |  |  |  |  |  | 0 | 30 |  |  |  |  |  |  | 50 |  |  |  |  |  |  |  |  | 10 |
| CDDP | lower | 180 |  | -5 |  |  |  |  |  |  |  |  |  |  |  |  | -100 |  |  |  |  |  |  |  |  |  |
| CDDP | upper | 365 |  | 30 |  |  |  |  |  |  |  |  |  |  |  |  | 1000 |  |  |  |  |  |  |  |  |  |
| CDDP_CO_2_ | lower | 180 |  | -5 |  |  |  |  |  |  |  |  |  |  |  |  | -100 |  |  |  |  |  |  |  |  | -100 |
| CDDP_CO_2_ | upper | 365 |  | 30 |  |  |  |  |  |  |  |  |  |  |  |  | 1000 |  |  |  |  |  |  |  |  | 250 |
| CDDM | lower | 180 |  | -5 |  |  |  |  | -5 |  |  |  |  |  |  |  | -1000 |  |  |  |  |  |  |  |  |  |
| CDDM | upper | 365 |  | 30 |  |  |  |  | 5 |  |  |  |  |  |  |  | 1000 |  |  |  |  |  |  |  |  |  |
| CDDM_CO_2_ | lower | 180 |  | -5 |  |  |  |  | -5 |  |  |  |  |  |  |  | -1000 |  |  |  |  |  |  |  |  | -250 |
| CDDM_CO_2_ | upper | 365 |  | 30 |  |  |  |  | 5 |  |  |  |  |  |  |  | 1000 |  |  |  |  |  |  |  |  | 250 |
| PPM | lower | 180 |  |  |  |  |  |  | -5 |  |  |  |  |  |  |  | -100 |  |  |  |  |  |  |  |  |  |
| PPM | upper | 365 |  |  |  |  |  |  | 5 |  |  |  |  |  |  |  | 100 |  |  |  |  |  |  |  |  |  |
| PPM_CO_2_ | lower | 180 |  |  |  |  |  |  | -5 |  |  |  |  |  |  |  | -100 |  |  |  |  |  |  |  |  | -25 |
| PPM_CO_2_ | upper | 365 |  |  |  |  |  |  | 5 |  |  |  |  |  |  |  | 100 |  |  |  |  |  |  |  |  | 25 |

**TABLE S2** Lowest AIC, mean AIC and standard deviation of AIC, lowest RMSE, mean RMSE, and standard deviation of RMSE of 25 parallel chains for all spring models using 2m air temperature for larch, spruce, and shrub. Model details are listed in Table 1. Bold numbers refer to the best models (∆AIC<2) per plant functional type as shown in Figure 4.

***** Depth refers to air or soil temperature dataset for the best model only

| **Model** | **Lowest AIC** | **Mean AIC** | **SD AIC** | **Lowest RMSE** | **Mean RMSE** | **SD RMSE** | **Depth (cm)*** |
| --- | --- | --- | --- | --- | --- | --- | --- |
| ***Larch*** |  |  |  |  |  |  |  |
| **SM1b** | **117.4** | **124.0** | **4.7** | **3.3** | **3.3** | **0.2** | **air** |
| **PTT** | **117.5** | **118.3** | **1.8** | **3.5** | **3.5** | **0.1** | **air** |
| **TTs** | **118.1** | **123.9** | **5.3** | **3.6** | **3.6** | **0.2** | **air** |
| **TT** | **118.2** | **118.7** | **0.7** | **3.5** | **3.5** | **0.0** | **air** |
| **PTTs** | **118.6** | **123.6** | **6.0** | **3.6** | **3.6** | **0.3** | **air** |
| **SM1** | **118.6** | **127.7** | **5.6** | **3.5** | **3.5** | **0.2** | **air** |
| **M1** | **119** | **121.0** | **2.0** | **3.5** | **3.5** | **0.1** | **air** |
| UM1 | 120.7 | 134.1 | 8.1 | 3.7 | 3.7 | 0.3 | air |
| PA | 121.6 | 128.0 | 2.8 | 3.4 | 3.4 | 0.1 | air |
| AT | 122.2 | 123.2 | 1.3 | 3.5 | 3.5 | 0.0 | air |
| PAb | 122.4 | 128.0 | 3.3 | 3.4 | 3.4 | 0.1 | air |
| M1s | 122.6 | 131.5 | 5.7 | 3.9 | 3.9 | 0.2 | air |
| PM1b | 127.1 | 128.0 | 1.1 | 3.5 | 3.5 | 0.0 | air |
| PM1 | 127.2 | 128.5 | 1.5 | 3.5 | 3.5 | 0.1 | air |
| SQ | 128.2 | 131.0 | 2.8 | 3.6 | 3.6 | 0.1 | air |
| SQb | 128.2 | 130.9 | 2.7 | 3.6 | 3.6 | 0.1 | air |
| AGSI | 129.8 | 142.0 | 1.8 | 3.7 | 4.1 | 0.1 | 0 |
| LIN | 149 | 170.7 | 0.0 | 5.0 | 6.4 | 0.0 | 30 |
| SGSI | 193.6 | 214.1 | 8.7 | 9.0 | 9.3 | 0.9 | 0 |
| ***Spruce*** |  |  |  |  |  |  |  |
| **SM1** | **161.5** | **194.0** | **5.9** | **7.5** | **7.1** | **0.4** | **air** |
| **SM1b** | **162.2** | **195.5** | **0.2** | **7.5** | **7.5** | **0.0** | **air** |
| PAb | 168.5 | 218.2 | 0.0 | 6.6 | 10.3 | 0.0 | air |
| UM1 | 169.8 | 189.9 | 3.2 | 9.6 | 7.2 | 0.3 | 20 |
| PA | 174.7 | 194.6 | 8.9 | 5.9 | 7.5 | 0.7 | 100 |
| AGSI | 177.9 | 187.0 | 8.2 | 4.9 | 6.3 | 0.6 | air |
| M1s | 183.0 | 180.1 | 7.7 | 5.5 | 5.8 | 0.5 | air |
| SGSI | 185.7 | 201.1 | 0.3 | 7.2 | 7.5 | 0.0 | air |
| M1 | 186.1 | 200.8 | 0.9 | 5.3 | 7.5 | 0.1 | air |
| PTTs | 188.1 | 191.0 | 0.3 | 7.3 | 7.5 | 0.0 | air |
| TT | 188.5 | 192.5 | 3.6 | 7.3 | 7.4 | 0.3 | air |
| TTs | 189.3 | 202.6 | 6.0 | 7.5 | 7.8 | 0.5 | air |
| PTT | 190.8 | 191.8 | 11.9 | 7.1 | 6.8 | 0.8 | air |
| AT | 195.4 | 183.3 | 10.9 | 4.9 | 6.2 | 0.7 | air |
| PM1b | 197.6 | 201.7 | 1.4 | 6.9 | 7.5 | 0.1 | air |
| SQ | 198.5 | 202.1 | 1.6 | 7.2 | 7.6 | 0.1 | air |
| SQb | 198.5 | 191.4 | 0.6 | 7.3 | 7.5 | 0.1 | air |
| PM1 | 200.8 | 192.6 | 2.3 | 6.5 | 7.4 | 0.2 | air |
| LIN | 211.7 | 178.9 | 9.1 | 5.1 | 5.9 | 0.6 | air |
|  |  |  |  |  |  |  |  |
| ***Shrub*** |  |  |  |  |  |  |  |
| **PAb** | **145.6** | **172.4** | **4.3** | **3.7** | **5.0** | **0.2** | **air** |
| **PA** | **147.0** | **187.4** | **0.2** | **3.7** | **6.1** | **0.0** | **air** |
| AGSI | 159.9 | 186.6 | 0.0 | 4.4 | 6.4 | 0.0 | 0 |
| TTs | 160.0 | 183.2 | 0.6 | 4.7 | 6.0 | 0.0 | air |
| PTTs | 161.2 | 168.5 | 3.1 | 4.8 | 5.0 | 0.2 | air |
| SGSI | 162.8 | 161.8 | 14.9 | 4.6 | 4.4 | 0.7 | air |
| M1s | 163.6 | 170.8 | 10.7 | 4.8 | 4.8 | 0.5 | air |
| SM1b | 165.7 | 193.0 | 1.4 | 4.6 | 6.1 | 0.1 | 10 |
| UM1 | 166.0 | 193.8 | 1.9 | 4.6 | 6.1 | 0.1 | air |
| SM1 | 167.4 | 182.7 | 0.7 | 4.7 | 6.1 | 0.0 | 40 |
| LIN | 170.5 | 162.1 | 0.9 | 5.5 | 4.8 | 0.0 | 40 |
| PTT | 176.8 | 175.9 | 10.5 | 5.7 | 5.2 | 0.6 | 50 |
| TT | 177.2 | 179.3 | 6.0 | 5.7 | 5.3 | 0.3 | 50 |
| M1 | 178.8 | 181.0 | 5.9 | 5.7 | 5.4 | 0.3 | 50 |
| AT | 181.0 | 196.2 | 2.0 | 5.7 | 6.3 | 0.1 | 50 |
| PM1 | 186.8 | 196.3 | 2.4 | 5.7 | 6.3 | 0.2 | 50 |
| PM1b | 186.8 | 183.7 | 0.8 | 5.7 | 6.1 | 0.1 | 50 |
| SQ | 187.2 | 161.8 | 1.4 | 5.7 | 4.8 | 0.1 | 50 |
| SQb | 187.2 | 193.2 | 8.2 | 5.7 | 6.1 | 0.5 | 50 |

**TABLE S3** Mean AIC and standard deviation of 25 parallel model chains for all autumn models and three plant functional types. Model details are listed in Table 1. Bold models refer to the best model as shown in Figure 5.

| **Model** | **Lowest AIC** | **Mean AIC** | **SD AIC** | **Lowest RMSE** | **Mean RMSE** | **SD RMSE** | **Depth (cm)*** |
| --- | --- | --- | --- | --- | --- | --- | --- |
| ***Larch*** |  |  |  |  |  |  |  |
| **CDDs_CO_2_** | **133.2** | **141.6** | **1.0** | **3.9** | **4.3** | **0.0** | **air** |
| CDD_CO_2_ | 137.3 | 138.6 | 0.8 | 4.2 | 4.3 | 0.0 | air |
| CDDP_CO_2_ | 138.0 | 140.0 | 0.8 | 4.2 | 4.3 | 0.0 | air |
| CDDM_CO_2_ | 140.2 | 135.5 | 1.5 | 4.3 | 4.0 | 0.1 | air |
| CDDM | 166.5 | 168.4 | 0.4 | 5.8 | 5.9 | 0.0 | 200 |
| CDDP | 167.2 | 168.2 | 0.8 | 6.0 | 6.1 | 0.1 | air |
| CDDs | 168.0 | 169.2 | 0.4 | 5.9 | 6.0 | 0.0 | air |
| CDD | 168.7 | 170.6 | 0.2 | 6.1 | 6.2 | 0.0 | 100 |
| PPM_CO_2_ | 188.7 | 189.5 | 0.5 | 7.5 | 7.5 | 0.0 | 10 |
| PPM | 192.6 | 192.9 | 0.4 | 7.9 | 8.0 | 0.0 | 50 |
| ***Spruce*** |  |  |  |  |  |  |  |
| **CDD_CO_2_** | **179.6** | **186.1** | **0.4** | **5.6** | **5.9** | **0.0** | **5** |
| CDDP_CO_2_ | 183.6 | 192.4 | 0.5 | 5.8 | 6.3 | 0.0 | 10 |
| CDDs_CO_2_ | 184.3 | 195.3 | 3.0 | 5.7 | 6.4 | 0.2 | 10 |
| CDD | 188.4 | 188.7 | 0.2 | 8.1 | 6.2 | 0.0 | 200 |
| CDDM_CO_2_ | 189.5 | 194.2 | 0.9 | 6.0 | 6.3 | 0.1 | 30 |
| CDDP | 193.5 | 194.0 | 0.5 | 6.5 | 6.6 | 0.0 | air |
| CDDs | 194.7 | 200.1 | 2.0 | 6.5 | 6.8 | 0.1 | air |
| CDDM | 197.9 | 196.2 | 1.1 | 6.7 | 6.6 | 0.1 | air |
| PPM_CO_2_ | 216.7 | 218.0 | 0.8 | 8.1 | 8.2 | 0.1 | 30 |
| PPM | 220.5 | 220.7 | 0.5 | 8.5 | 8.6 | 0.0 | air |
| ***Shrub*** |  |  |  |  |  |  |  |
| **CDD_CO_2_** | **213.2** | **217.6** | **0.4** | **8.1** | **8.5** | **0.0** | **0** |
| CDD | 215.3 | 215.9 | 0.5 | 8.5 | 8.5 | 0.0 | air |
| CDDP_CO_2_ | 216.4 | 219.2 | 0.9 | 8.4 | 8.6 | 0.1 | 10 |
| CDDM | 216.4 | 218.3 | 0.5 | 8.4 | 8.5 | 0.0 | air |
| CDDP | 217.3 | 217.8 | 0.5 | 8.6 | 8.7 | 0.0 | air |
| CDDs | 217.4 | 217.7 | 1.0 | 8.5 | 8.5 | 0.1 | air |
| CDDM_CO_2_ | 217.7 | 219.8 | 0.5 | 8.3 | 8.5 | 0.0 | 30 |
| CDDs_CO_2_ | 218.0 | 219.2 | 0.8 | 8.4 | 8.5 | 0.1 | 0 |
| PPM | 249.3 | 249.7 | 0.3 | 12.0 | 12.0 | 0.0 | 0 |
| PPM_CO_2_ | 251.2 | 251.8 | 0.4 | 12.0 | 12.0 | 0.0 | air |

***** Depth refers to temperature dataset for the best model only

**TABLE S4** Parameter values (Mean and SD) from 25 parallel model chains for spring and autumn models for *Larix* (DN), *Spruce* (EN), and shrub (SH) using air temperature.

| **Model** | **PFT** | **Mean** | **SD** | **Mean** | **SD** | **Mean** | **SD** | **Mean** | **SD** | **Mean** | **SD** | **Mean** | **SD** | **Mean** | **SD** | **Mean** | **SD** | **Mean** | **SD** |
| --- | --- | --- | --- | --- | --- | --- | --- | --- | --- | --- | --- | --- | --- | --- | --- | --- | --- | --- | --- |
| **LIN** |  | **a** |  | **b** |  |  |  |  |  |  |  |  |  |  |  |  |  |  |  |
|  | DN | -1.5 | 0.0 | 127.7 | 0.0 |  |  |  |  |  |  |  |  |  |  |  |  |  |  |
|  | EN | -1.8 | 0.0 | 104.4 | 0.0 |  |  |  |  |  |  |  |  |  |  |  |  |  |  |
|  | SH | -0.9 | 0.0 | 144.8 | 0.0 |  |  |  |  |  |  |  |  |  |  |  |  |  |  |
| **TT** |  | **t0** |  | **T_base** |  | **F_crit** |  |  |  |  |  |  |  |  |  |  |  |  |  |
|  | DN | 88.1 | 0.7 | 2.5 | 0.8 | 146.8 | 18.8 |  |  |  |  |  |  |  |  |  |  |  |  |
|  | EN | 66.4 | 2.3 | -1.0 | 2.3 | 105.1 | 30.5 |  |  |  |  |  |  |  |  |  |  |  |  |
|  | SH | 90.9 | 8.1 | -4.3 | 2.8 | 724.4 | 145.5 |  |  |  |  |  |  |  |  |  |  |  |  |
| **TTs** |  | **t0** |  | **a** |  | **b** |  | **F_crit** |  |  |  |  |  |  |  |  |  |  |  |
|  | DN | 87.2 | 4.0 | 0.3 | 0.1 | 10.7 | 3.0 | 9.8 | 4.7 |  |  |  |  |  |  |  |  |  |  |
|  | EN | 63.4 | 19.2 | 6.7 | 14.8 | 7.2 | 2.2 | 7.1 | 8.9 |  |  |  |  |  |  |  |  |  |  |
|  | SH | 86.9 | 3.4 | 2.7 | 5.2 | 3.0 | 1.1 | 42.9 | 4.3 |  |  |  |  |  |  |  |  |  |  |
| **PTT** |  | **t0** |  | **T_base** |  | **F_crit** |  |  |  |  |  |  |  |  |  |  |  |  |  |
|  | DN | 87.2 | 1.8 | 2.6 | 1.2 | 83.2 | 21.9 |  |  |  |  |  |  |  |  |  |  |  |  |
|  | EN | 65.1 | 0.5 | -1.5 | 0.5 | 53.2 | 4.3 |  |  |  |  |  |  |  |  |  |  |  |  |
|  | SH | 89.2 | 1.8 | -4.8 | 0.2 | 437.7 | 16.0 |  |  |  |  |  |  |  |  |  |  |  |  |
| **PTTs** |  | **t0** |  | **b** |  | **c** |  | **F_crit** |  |  |  |  |  |  |  |  |  |  |  |
|  | DN | 85.9 | 11.3 | 14.7 | 33.6 | 10.0 | 3.0 | 6.0 | 3.6 |  |  |  |  |  |  |  |  |  |  |
|  | EN | 55.0 | 37.2 | 24.2 | 34.6 | 6.5 | 3.0 | 5.1 | 7.3 |  |  |  |  |  |  |  |  |  |  |
|  | SH | 88.8 | 1.3 | 42.6 | 33.9 | 4.7 | 1.1 | 23.1 | 1.4 |  |  |  |  |  |  |  |  |  |  |
| **M1** |  | **t0** |  | **T_base** |  | **f** |  | **F_crit** |  |  |  |  |  |  |  |  |  |  |  |
|  | DN | 86.1 | 5.6 | 2.8 | 1.1 | 2.9 | 1.5 | 391.3 | 232.4 |  |  |  |  |  |  |  |  |  |  |
|  | EN | 64.9 | 7.8 | 2.2 | 2.8 | 4.3 | 0.8 | 123.8 | 98.1 |  |  |  |  |  |  |  |  |  |  |
|  | SH | 88.9 | 1.1 | -4.8 | 0.2 | 2.3 | 0.6 | 1656.3 | 295.8 |  |  |  |  |  |  |  |  |  |  |
| **M1s** |  | **t0** |  | **b** |  | **c** |  | **f** |  | **F_crit** |  |  |  |  |  |  |  |  |  |
|  | DN | 27.7 | 63.3 | 2.0 | 8.5 | 7.9 | 5.0 | 4.4 | 0.8 | 71.5 | 42.5 |  |  |  |  |  |  |  |  |
|  | EN | 19.1 | 58.8 | 24.2 | 35.6 | 4.3 | 3.2 | 3.5 | 1.9 | 34.2 | 30.9 |  |  |  |  |  |  |  |  |
|  | SH | 72.9 | 36.0 | 53.7 | 37.4 | 4.3 | 1.7 | 3.1 | 1.4 | 133.6 | 61.7 |  |  |  |  |  |  |  |  |
| **AT** |  | **t0** |  | **T_base** |  | **a** |  | **b** |  | **c** |  |  |  |  |  |  |  |  |  |
|  | DN | 87.8 | 1.1 | 1.7 | 1.2 | 77.1 | 51.5 | 88.7 | 60.2 | 2.1 | 1.4 |  |  |  |  |  |  |  |  |
|  | EN | 65.8 | 0.5 | -1.8 | 0.3 | 56.0 | 41.2 | 59.4 | 40.5 | 2.8 | 1.4 |  |  |  |  |  |  |  |  |
|  | SH | 87.8 | 0.5 | -4.8 | 0.1 | 265.7 | 143.4 | 491.9 | 146.1 | 3.0 | 1.5 |  |  |  |  |  |  |  |  |
| **SQ** |  | **t0** |  | **t0_chill** |  | **T_base** |  | **T_opt** |  | **T_min** |  | **T_max** |  | **F_crit** |  | **C_req** |  |  |  |
|  | DN | 87.3 | 3.0 | 51.1 | 43.7 | 1.4 | 2.6 | 3.8 | 2.8 | -2.1 | 1.9 | 10.1 | 2.6 | 181.9 | 83.1 | 8.8 | 5.9 |  |  |
|  | EN | 66.7 | 2.8 | 34.9 | 22.1 | -0.7 | 3.1 | 2.3 | 2.9 | -2.4 | 2.0 | 10.0 | 3.5 | 102.1 | 44.1 | 8.8 | 8.0 |  |  |
|  | SH | 111.9 | 18.9 | 50.0 | 32.3 | 1.2 | 6.4 | 2.7 | 3.1 | -2.0 | 2.4 | 9.8 | 4.0 | 358.9 | 338.5 | 10.2 | 5.8 |  |  |
| **SQb** |  | **t0** |  | **t0_chill** |  | **T_base** |  | **a** |  | **b** |  | **c** |  | **F_crit** |  | **C_req** |  |  |  |
|  | DN | 88.1 | 2.6 | 48.3 | 32.0 | 2.2 | 2.7 | 2.9 | 2.8 | -2.1 | 13.6 | -1.7 | 5.1 | 160.7 | 79.0 | 13.9 | 21.4 |  |  |
|  | EN | 67.1 | 3.1 | 51.9 | 35.1 | -0.3 | 3.3 | 2.0 | 2.1 | 5.0 | 14.0 | -0.1 | 4.9 | 95.7 | 46.5 | 13.5 | 18.6 |  |  |
|  | SH | 110.1 | 18.8 | 49.9 | 33.3 | -0.7 | 4.8 | 3.4 | 2.7 | -0.6 | 14.6 | -0.9 | 3.6 | 405.3 | 325.3 | 9.5 | 12.7 |  |  |
| **SM1** |  | **t0** |  | **t0_chill** |  | **T_base** |  | **T_opt** |  | **T_min** |  | **T_max** |  | **F_crit** |  | **C_req** |  |  |  |
|  | DN | 86.3 | 3.2 | 90.6 | 56.8 | 0.2 | 2.5 | 4.5 | 4.2 | 1.1 | 4.5 | 11.0 | 4.2 | 216.8 | 86.3 | 101.5 | 97.9 |  |  |
|  | EN | 66.9 | 4.9 | 87.4 | 56.9 | -0.3 | 2.6 | 3.5 | 2.8 | -0.9 | 3.7 | 9.5 | 3.6 | 85.3 | 45.8 | 40.8 | 57.5 |  |  |
|  | SH | 105.3 | 14.1 | 114.8 | 53.1 | -3.5 | 1.9 | 4.4 | 3.8 | -1.5 | 2.7 | 10.8 | 3.0 | 435.7 | 192.1 | 26.6 | 13.0 |  |  |
| **SM1b** |  | **t0** |  | **t0_chill** |  | **T_base** |  | **a** |  | **b** |  | **c** |  | **F_crit** |  | **C_req** |  |  |  |
|  | DN | 87.4 | 2.8 | 188.4 | 2.8 | 1.5 | 2.4 | 5.1 | 2.7 | -4.8 | 12.6 | 4.8 | 5.1 | 171.3 | 65.1 | 64.3 | 98.5 |  |  |
|  | EN | 65.3 | 4.5 | 79.3 | 53.8 | -0.1 | 2.4 | 3.8 | 2.6 | -2.0 | 13.0 | 3.0 | 4.7 | 81.6 | 43.8 | 30.8 | 58.2 |  |  |
|  | SH | 107.0 | 15.8 | 103.6 | 55.8 | -3.5 | 1.5 | 2.8 | 1.9 | -2.9 | 13.8 | 2.2 | 5.5 | 429.6 | 233.2 | 27.9 | 19.0 |  |  |
| **PA** |  | **t0** |  | **t0_chill** |  | **T_base** |  | **T_opt** |  | **T_min** |  | **T_max** |  | **C_ini** |  | **F_crit** |  | **C_req** |  |
|  | DN | 85.9 | 5.5 | 154.4 | 78.9 | 1.5 | 2.6 | 3.0 | 3.9 | -1.5 | 3.0 | 7.7 | 4.0 | 0.4 | 0.3 | 136.7 | 106.7 | 64.9 | 75.7 |
|  | EN | 59.3 | 12.7 | 139.4 | 46.3 | 0.3 | 3.7 | -0.1 | 3.5 | -2.9 | 2.9 | 5.0 | 3.8 | 0.2 | 0.2 | 73.2 | 76.6 | 13.2 | 16.6 |
|  | SH | 80.4 | 33.9 | 190.0 | 58.9 | -1.4 | 3.5 | 5.3 | 2.9 | 0.1 | 3.3 | 11.4 | 2.1 | 0.3 | 0.2 | 245.5 | 167.1 | 16.8 | 24.0 |
| **PAb** |  | **t0** |  | **t0_chill** |  | **T_base** |  | **a** |  | **b** |  | **c** |  | **C_ini** |  | **F_crit** |  | **C_req** |  |
|  | DN | 85.6 | 7.2 | 112.5 | 64.6 | 2.0 | 2.7 | 3.8 | 2.6 | -1.0 | 12.9 | 1.4 | 4.3 | 0.5 | 0.2 | 113.2 | 94.9 | 105.6 | 97.3 |
|  | EN | 54.5 | 23.8 | 98.7 | 44.6 | -0.7 | 2.7 | 3.1 | 2.8 | 3.5 | 15.0 | -2.4 | 3.3 | 0.1 | 0.1 | 80.0 | 59.6 | 39.8 | 37.6 |
|  | SH | 91.0 | 14.6 | 96.9 | 80.6 | 96.9 | 80.6 | 3.2 | 2.1 | 2.6 | 17.0 | -2.0 | 6.2 | 0.4 | 0.2 | 362.1 | 207.0 | 60.0 | 65.2 |
| **PM1** |  | **t0** |  | **t0_chill** |  | **T_base** |  | **T_opt** |  | **T_min** |  | **T_max** |  | **C_ini** |  | **F_crit** |  |  |  |
|  | DN | 87.9 | 1.2 | 188.9 | 1.2 | 2.2 | 5.3 | 3.3 | 4.9 | 7.3 | 3.4 | 0.6 | 0.3 | 188.4 | 53.0 | 171.0 | 92.9 |  |  |
|  | EN | 66.0 | 0.6 | -1.4 | 0.6 | 2.6 | 5.2 | 2.1 | 4.0 | 7.5 | 5.3 | 0.8 | 0.2 | 121.4 | 12.2 | 165.0 | 119.5 |  |  |
|  | SH | 86.6 | 11.2 | -4.2 | 2.8 | 3.0 | 4.6 | 3.2 | 4.4 | 8.0 | 4.5 | 0.7 | 0.3 | 893.1 | 255.4 | 184.6 | 109.7 |  |  |
| **PM1b** |  | **t0** |  | **T_base** |  | **a** |  | **b** |  | **c** |  | **C_ini** |  | **F_crit** |  | **C_req** |  |  |  |
|  | DN | 87.5 | 0.8 | 2.3 | 1.1 | 5.7 | 2.5 | 1.6 | 12.4 | 0.8 | 5.8 | 0.6 | 0.3 | 182.4 | 45.1 | 161.5 | 88.9 |  |  |
|  | EN | 66.2 | 2.5 | -0.6 | 2.2 | 5.8 | 3.1 | 3.0 | 10.3 | 3.0 | 10.3 | 0.9 | 0.1 | 110.1 | 33.7 | 110.1 | 33.7 |  |  |
|  | SH | 98.8 | 17.3 | -1.8 | 5.7 | 5.8 | 2.9 | -2.2 | 10.3 | -1.3 | 5.4 | 0.6 | 0.2 | 718.3 | 386.0 | 165.9 | 96.3 |  |  |
| **UM1** |  | **t0** |  | **T_base** |  | **T_opt** |  | **T_min** |  | **T_max** |  | **f** |  | **w** |  | **C_req** |  |  |  |
|  | DN | 93.1 | 3.4 | 8.7 | 1.5 | 5.2 | 4.0 | 4.1 | 3.6 | 6.7 | 4.8 | -2.2 | 4.3 | 50.7 | 21.8 | 128.5 | 103.8 |  |  |
|  | EN | 69.2 | 7.8 | 8.5 | 0.9 | 3.1 | 2.2 | 1.5 | 1.5 | 5.0 | 3.1 | -13.9 | 23.5 | 91.4 | 212.1 | 150.7 | 103.8 |  |  |
|  | SH | 123.1 | 11.9 | 6.2 | 4.1 | 4.7 | 4.3 | 1.8 | 4.5 | 7.6 | 3.7 | -8.9 | 20.2 | 150.1 | 213.1 | 157.7 | 106.7 |  |  |
| **SGSI** |  | **T_min** |  | **T_max** |  | **F_crit** |  | **VPD_min** |  | **VPD_max** |  | **L_min** |  | **L_max** |  |  |  |  |  |
|  | DN | -8.9 | 4.8 | 6.4 | 6.5 | 0.6 | 0.4 | 1224.3 | 740.7 | 3292.7 | 717.8 | 11.4 | 0.3 | 12.0 | 0.1 |  |  |  |  |
|  | EN | -12.6 | 2.9 | 5.9 | 6.3 | 0.5 | 0.2 | 1185.9 | 565.1 | 3227.8 | 869.0 | 11.2 | 0.3 | 11.9 | 0.1 |  |  |  |  |
|  | SH | -13.5 | 3.0 | 6.4 | 5.9 | 0.1 | 0.0 | 147.4 | 24.1 | 4261.4 | 661.1 | 10.1 | 1.0 | 11.3 | 0.5 |  |  |  |  |
| **AGSI** |  | **T_min** |  | **T_max** |  | **F_crit** |  | **VPD_min** |  | **VPD_max** |  | **L_min** |  | **L_max** |  |  |  |  |  |
|  | DN | -13.2 | 1.1 | 12.9 | 5.3 | 20.6 | 4.1 | 1474.7 | 334.3 | 3539.3 | 862.8 | 11.3 | 0.3 | 11.9 | 0.1 |  |  |  |  |
|  | EN | -9.8 | 2.3 | 12.2 | 8.2 | 7.6 | 5.5 | 1131.1 | 663.2 | 3498.8 | 951.2 | 10.0 | 1.2 | 11.3 | 0.5 |  |  |  |  |
|  | SH | -14.6 | 0.7 | 2.0 | 3.2 | 51.8 | 10.0 | 1665.0 | 379.7 | 3464.7 | 937.1 | 11.5 | 0.1 | 11.9 | 0.1 |  |  |  |  |
| **CDD** |  | **t0** |  | **T_base** |  | **F_crit** |  |  |  |  |  |  |  |  |  |  |  |  |  |
|  | DN | 242.2 | 3.5 | 35.9 | 1.4 | -858.0 | 93.7 |  |  |  |  |  |  |  |  |  |  |  |  |
|  | EN | 266.3 | 2.1 | 28.9 | 1.1 | -802.8 | 67.1 |  |  |  |  |  |  |  |  |  |  |  |  |
|  | SH | 247.8 | 2.4 | 21.7 | 1.1 | -319.6 | 48.0 |  |  |  |  |  |  |  |  |  |  |  |  |
| **CDD_CO_2_** |  | **t0** |  | **T_base** |  | **F_crit** |  | **CO_2_** |  |  |  |  |  |  |  |  |  |  |  |
|  | DN | 244.3 | 2.5 | 32.7 | 1.6 | -764.3 | 104.1 | -189.3 | 17.5 |  |  |  |  |  |  |  |  |  |  |
|  | EN | 269.5 | 2.8 | 28.6 | 1.6 | -800.0 | 104.2 | -80.8 | 10.5 |  |  |  |  |  |  |  |  |  |  |
|  | SH | 243.0 | 2.6 | 23.1 | 1.1 | -422.6 | 62.9 | -46.4 | 17.5 |  |  |  |  |  |  |  |  |  |  |
| **CDDs** |  | **t0** |  | **b** |  | **c** |  | **F_crit** |  |  |  |  |  |  |  |  |  |  |  |
|  | DN | 244.1 | 6.0 | -1.1 | 0.6 | 17.7 | 1.3 | 37.5 | 5.6 |  |  |  |  |  |  |  |  |  |  |
|  | EN | 267.7 | 6.1 | -0.2 | 0.1 | 6.9 | 3.4 | 23.7 | 5.7 |  |  |  |  |  |  |  |  |  |  |
|  | SH | 249.3 | 3.1 | -0.5 | 0.6 | 5.5 | 2.9 | 15.4 | 5.9 |  |  |  |  |  |  |  |  |  |  |
| **CDDs_CO_2_** |  | **t0** |  | **b** |  | **c** |  | **F_crit** |  | **CO_2_** |  |  |  |  |  |  |  |  |  |
|  | DN | 242.9 | 5.3 | -0.6 | 0.3 | 16.6 | 1.3 | 38.7 | 5.6 | 8.7 | 0.8 |  |  |  |  |  |  |  |  |
|  | EN | 268.8 | 6.7 | -0.2 | 0.1 | 8.8 | 2.9 | 27.8 | 6.1 | 2.8 | 0.7 |  |  |  |  |  |  |  |  |
|  | SH | 249.1 | 2.6 | -0.6 | 0.5 | 8.1 | 2.0 | 21.4 | 5.0 | 1.8 | 0.7 |  |  |  |  |  |  |  |  |
| **CDDP** |  | **t0** |  | **T_base** |  | **F_crit** |  |  |  |  |  |  |  |  |  |  |  |  |  |
|  | DN | 272.1 | 3.0 | 18.3 | 2.1 | 71.5 | 27.9 |  |  |  |  |  |  |  |  |  |  |  |  |
|  | EN | 266.4 | 3.7 | 25.8 | 2.1 | 343.8 | 55.6 |  |  |  |  |  |  |  |  |  |  |  |  |
|  | SH | 250.4 | 1.7 | 18.6 | 1.8 | 174.0 | 43.9 |  |  |  |  |  |  |  |  |  |  |  |  |
| **CDDP_CO_2_** |  | **t0** |  | **T_base** |  | **F_crit** |  | **CO_2_** |  |  |  |  |  |  |  |  |  |  |  |
|  | DN | 251.6 | 5.2 | 26.2 | 2.8 | 276.6 | 79.6 | 66.6 | 10.7 |  |  |  |  |  |  |  |  |  |  |
|  | EN | 267.3 | 5.3 | 25.9 | 2.8 | 359.5 | 72.0 | 31.6 | 6.6 |  |  |  |  |  |  |  |  |  |  |
|  | SH | 249.9 | 2.3 | 19.0 | 2.3 | 191.1 | 55.0 | 11.6 | 4.7 |  |  |  |  |  |  |  |  |  |  |
| **CDDM** |  | **t0** |  | **T_base** |  | **b** |  | **F_crit** |  |  |  |  |  |  |  |  |  |  |  |
|  | DN | 272.0 | 4.5 | 18.0 | 2.7 | 1.2 | 0.5 | -162.9 | 89.5 |  |  |  |  |  |  |  |  |  |  |
|  | EN | 270.9 | 8.3 | 23.4 | 5.1 | 1.7 | 0.6 | -671.3 | 193.2 |  |  |  |  |  |  |  |  |  |  |
|  | SH | 258.4 | 5.2 | 16.7 | 3.8 | 2.3 | 0.4 | -309.4 | 97.7 |  |  |  |  |  |  |  |  |  |  |
| **CDDM_CO_2_** |  | **t0** |  | **T_base** |  | **b** |  | **F_crit** |  | **CO_2_** |  |  |  |  |  |  |  |  |  |
|  | DN | 260.6 | 6.1 | 20.2 | 2.9 | -0.3 | 0.4 | -345.9 | 118.3 | -118.2 | 29.9 |  |  |  |  |  |  |  |  |
|  | EN | 273.5 | 7.1 | 21.7 | 3.2 | 1.1 | 0.5 | -637.8 | 183.9 | -83.0 | 21.9 |  |  |  |  |  |  |  |  |
|  | SH | 258.0 | 3.2 | 18.1 | 2.5 | 2.4 | 0.3 | -354.8 | 92.3 | -19.4 | 8.1 |  |  |  |  |  |  |  |  |
| **PPM** |  | **t0** |  | **b** |  | **F_crit** |  |  |  |  |  |  |  |  |  |  |  |  |  |
|  | DN | 263.0 | 4.9 | 2.5 | 0.4 | 25.6 | 4.7 |  |  |  |  |  |  |  |  |  |  |  |  |
|  | EN | 306.7 | 4.2 | 5.0 | 0.1 | 5.0 | 2.9 |  |  |  |  |  |  |  |  |  |  |  |  |
|  | SH | 273.2 | 11.1 | 4.3 | 0.6 | 24.4 | 9.8 |  |  |  |  |  |  |  |  |  |  |  |  |
| **PPM_CO_2_** |  | **t0** |  | **b** |  | **F_crit** |  | **CO_2_** |  |  |  |  |  |  |  |  |  |  |  |
|  | DN | 262.3 | 5.1 | 2.0 | 0.4 | 29.5 | 5.1 | 5.4 | 0.7 |  |  |  |  |  |  |  |  |  |  |
|  | EN | 291.4 | 10.4 | 3.8 | 0.8 | 20.1 | 10.0 | 4.1 | 0.9 |  |  |  |  |  |  |  |  |  |  |
|  | SH | 268.4 | 12.2 | 4.0 | 0.7 | 28.7 | 10.9 | 0.1 | 0.8 |  |  |  |  |  |  |  |  |  |  |

| **TABLE S5** Linear mixed effects model results for ground observations of larch to explain variation in autumn needle senescence. SE is standard error and DF degrees of freedom. |
| --- |
| \| Fixed effects \| Estimate \| SE \| Df \| t value \| P value \| \| --- \| --- \| --- \| --- \| --- \| --- \| \| Intercept \| 275.05 \| 9.0 \| 34 \| 30.68 \| <0.001 \| \| CO_2_ \| -9.61 \| 2.52 \| 34 \| -3.82 \| <0.001 \| \| Temperature \| 3.11 \| 0.4 \| 34 \| 7.82 \| <0.001 \| |
